# *Grxcr2* is required for stereocilia morphogenesis in the cochlea

**DOI:** 10.1371/journal.pone.0201713

**Published:** 2018-08-29

**Authors:** Matthew R. Avenarius, Jae-Yun Jung, Charles Askew, Sherri M. Jones, Kristina L. Hunker, Hela Azaiez, Atteeq U. Rehman, Margit Schraders, Hossein Najmabadi, Hannie Kremer, Richard J. H. Smith, Gwenaëlle S. G. Géléoc, David F. Dolan, Yehoash Raphael, David C. Kohrman

**Affiliations:** 1 Department of Otolaryngology/Kresge Hearing Research Institute, University of Michigan Medical School, Ann Arbor, Michigan, United States of America; 2 Department of Human Genetics, University of Michigan Medical School, Ann Arbor, Michigan, United States of America; 3 Neuroscience Graduate Program, University of Virginia, Charlottesville, Virginia, United States of America; 4 Boston Children’s Hospital, Harvard Medical School, Boston, Massachusetts, United States of America; 5 Department of Communication Sciences and Disorders, East Carolina University, Greenville, North Carolina, United States of America; 6 Molecular Otolaryngology and Renal Research Laboratories, Carver College of Medicine, University of Iowa, Iowa City, Iowa, United States of America; 7 Section on Human Genetics, Laboratory of Molecular Genetics, National Institute on Deafness and Other Communication Disorders, National Institutes of Health, Rockville, Maryland, United States of America; 8 Hearing & Genes Division, Department of Otorhinolaryngology, Radboud University Medical Center, Nijmegen, The Netherlands; 9 Donders Institute for Brain, Cognition and Behaviour, Radboud University Medical Center, Nijmegen, The Netherlands; 10 Department of Human Genetics, Radboud University Medical Center, Nijmegen, The Netherlands; 11 Genetics Research Center, University of Social Welfare and Rehabilitation Sciences, Tehran, Iran; University of South Florida, UNITED STATES

## Abstract

Hearing and balance depend upon the precise morphogenesis and mechanosensory function of stereocilia, the specialized structures on the apical surface of sensory hair cells in the inner ear. Previous studies of *Grxcr1* mutant mice indicated a critical role for this gene in control of stereocilia dimensions during development. In this study, we analyzed expression of the paralog *Grxcr2* in the mouse and evaluated auditory and vestibular function of strains carrying targeted mutations of the gene. Peak expression of *Grxcr2* occurs during early postnatal development of the inner ear and GRXCR2 is localized to stereocilia in both the cochlea and in vestibular organs. Homozygous *Grxcr2* deletion mutants exhibit significant hearing loss by 3 weeks of age that is associated with developmental defects in stereocilia bundle orientation and organization. Despite these bundle defects, the mechanotransduction apparatus assembles in relatively normal fashion as determined by whole cell electrophysiological evaluation and FM1-43 uptake. Although *Grxcr2* mutants do not exhibit overt vestibular dysfunction, evaluation of vestibular evoked potentials revealed subtle defects of the mutants in response to linear accelerations. In addition, reduced *Grxcr2* expression in a hypomorphic mutant strain is associated with progressive hearing loss and bundle defects. The stereocilia localization of GRXCR2, together with the bundle pathologies observed in the mutants, indicate that GRXCR2 plays an intrinsic role in bundle orientation, organization, and sensory function in the inner ear during development and at maturity.

## Introduction

Stereocilia are mechanosensory structures central to the perception of sound and vestibular stimuli and are located on the apical surface of sensory hair cells of the inner ear. As stereocilia bundles are deflected in response to auditory or vestibular stimuli, putative mechanosensory channel(s) in the membrane of the stereocilia tips open to depolarize the associated hair cell and alter neurotransmitter release at the base of the hair cell [1]. Auditory or vestibular nerve fibers in turn conduct associated action potentials to the central nervous system that are ultimately perceived as sound or head position. Bundles are composed of 50 to 300 individual stereocilia that are arranged in a highly organized, graded staircase configuration [2]. Analysis of mutant mouse models has defined a set of genes required for various aspects of the normal development of bundle morphology and function, including the final dimension of individual stereocilia, the organization of bundles, and the orientation of bundles with respect to the overall sensory organ [3, 4]. Mutations in many of these genes have been implicated as the basis for hearing loss in humans, including nonsyndromic and syndromic forms, indicating the clinical relevance of the genes and the evolutionary conservation of pathways necessary for normal stereocilia development and sensory function [5, 6].

*Grxcr1*, a gene required for establishing normal stereocilia diameter during development, is mutated in the mouse pirouette strain, which exhibits profound deafness and vestibular dysfunction [7]. Similarly, mutations in human *GRXCR1* underlie early onset hearing loss in pedigrees segregating defects at the DFNB25 locus [8]. *Grxcr1* encodes a cysteine-rich protein with sequence similarity in its central domain to the glutaredoxin family of proteins. GRXCR1 localizes to stereocilia in the cochlea and in vestibular organs of the inner ear, where it is likely to influence actin filament architecture in the stereocilia core [7].

In the present study, we have characterized a paralogous vertebrate gene, *Grxcr2*, and the effects on inner ear function of a targeted mutation at this locus. Similar to *Grxcr1*, *Grxcr2* is expressed in sensory hair cells in the cochlea and in vestibular organs and GRXCR2 is localized to stereocilia. Mice homozygous for a *Grxcr2* deletion mutation exhibit severe hearing loss associated with early postnatal defects in orientation and organization of stereocilia bundles in the cochlea. *Grxcr2* deletion mutants also exhibit electrophysiological defects in vestibular function but do not circle or show other overt behaviors that are exhibited by *Grxcr1* mutants and associated with more severe vestibular pathology. The cochlear and vestibular phenotypes in *Grxcr1* and *Grxcr2* mutants indicate different roles in the inner ear for each gene and suggest GRXCR1 and GRXCR2, while similar in sequence and localization, carry out distinct biological functions.

## Materials and methods

### Ethics statements

All mice were cared for in accordance with protocols approved by the Animal Care Committees of the University of Michigan (protocol number 08234) and the University of Virginia (Protocol number 3123). The human studies and consent procedures were approved by the Institutional Review Boards at the University of Social Welfare and Rehabilitation Sciences (Tehran, Iran), the University of Iowa (Iowa City, IA), the National Institutes of Health, USA (protocol IRB OH93-DC-0016), the National Centre of Excellence in Molecular Biology, University of the Punjab, Lahore, Pakistan, and the medical ethics committee of the Radboud University Nijmegen Medical Centre. Written informed consent was obtained from all participating subjects or, in the case of children, from their parents.

### Protein sequence analysis

GRXCR2 orthologous proteins were identified from annotated sequence databases at NCBI and at the Ensembl Genome Server: mouse (*Mus musculus*; NP_001028598), human (NP_001073985), opossum (*Monodelphis domestica*; XP_001369076), chicken (*Gallus gallus*; XP_001234901), and frog (*Xenopus tropicalis*; XP_002935322). The zebrafish Grxcr2 orthologous protein corresponds to an RT-PCR product amplified from whole embryo RNA (36 hour post-fertilization) using standard methods. RT-PCR primers (5'-GAGGAGCTCCAAAGGCAAC-3' and 5'-CTCTCTGCCGTCAGGGTTAC-3') were derived from an Ensembl *in silico* cDNA sequence (ENSDART00000075765). Multiple sequence alignments of orthologs and of mouse GRXCR1 (AAU84851) were made using CLUSTALW [9] with MacVector software (MacVector, Inc.; Cary, NC). Protein structural predictions were performed by comparisons of sequences and structures available at the Structural Classification of Proteins database [10] and the Protein Data Bank [11] using the Phyre^2^ and I-TASSER analysis platforms [12, 13]. These platforms use sequence conservation profiles based upon PSI-BLAST alignments [14] and secondary structure predictions using Psi-Pred [15], SSPro [16] and JNet [17] to identify structurally homologous proteins and to predict folding models. Based upon the significance of threading alignments and simulations, I-TASSER provides a confidence score for predicted models that is strongly correlated with traditional measures of similarity among known structures such as the root-mean-square deviation (RMSD), a measure of the average distance between backbone atoms of superimposed protein structures [18]. The structure of the cysteine (Cys)-rich zinc-binding domain of dnaJ/Hsp40 is based upon the NMR solution structure of this domain (1exk) in complex with zinc ions [19]. The structure of the Cys-rich domain of DNAJ3 (2ctt) was derived from unpublished data available at the Protein Data Bank. Structures were visualized using PyMOL Molecular Graphics System, Version 1.5.0.4 (Schrödinger, LLC). GRXCR-related proteins in non-vertebrate lineages were identified by BLASTP searches of non-redundant sequences in Genbank, using the full-length (254 amino acids) mouse GRXCR2 sequence as the query. Following removal of alternative isoforms encoded by the same genes in each species, the 55 highest scoring sequences derived from eight non-vertebrate phyla, along with mouse GRXCR1 (Q50H32.1) and GRXCR2 (Q3TYR5.1) sequences, were aligned with the Constraint-based Multiple Alignment Tool [20] available at the National Center for Biotechnology Information (NCBI), using the COBALT algorithm [21]. A phylogenetic tree was constructed from the multiple alignment using the neighbor-joining method [22]. Sequence analysis parameters: *BLASTP search*—expect threshold (10); word size (6); matrix (BLOSUM62); gap costs (existence 11, extension 1); compositional adjustment (conditional compositional score matrix). *COBALT multiple sequence alignment*—gap penalties (-11, -1); end-gap penalties (-5, -1); conserved domain database search algorithm (reverse position-specific BLAST). *Phylogenetic tree generation*—neighbor joining method using the Kimura distance matrix, with maximum sequence difference of 0.85.

### *Grxcr2* conditional allele

*Grxcr2* exon 1 and flanking DNA sequences (490 bp 5’ of exon 1 to 514 bp 3’ of exon 1) were amplified from BAC clone RP23-326N11 (derived from mouse strain C57BL/6J) using primers that included sequences for a proximal loxP site. This cassette was cloned into the vector PL451, which supplied the distal loxP site and neomycin selection gene [23]. Contiguous genomic DNA fragments extending 1,689 bp 5’ of this exon 1 fragment (5’ homology arm) and intron 1 extending 2,508 bp in the 3’ direction of the exon 1 fragment (3’ homology arm) were similarly amplified from RP23-326N11 and cloned into PL451. The resulting recombinant clone was digested with Sac II and Cla I to liberate the 7,534 bp targeting construct. The targeting construct was electroporated into the BRUCE4G9 C57BL/6 ES cell line [24] and Southern blot analyses were used to identify properly targeted clones. Three independent positive clones were microinjected into pseudopregnant females (*B6 Cg-Tyrc*^*2J*^) to derive chimeric mice that were subsequently mated to generate germ line founders (+/*Grxcr2*^*flox-neo*^). Founder mice were bred to mice homozygous for an EIIa-CRE recombinase transgene (B6.FVB-Tg(EIIa-cre)C5379Lmgd/J; Jackson Laboratories, stock number 003724), which drives Cre expression in the early mouse embryo [25]. Mice that inherited the floxed allele and the Cre transgene were evaluated for exon 1 deletion by genomic PCR. Carriers of the targeted mutant allele (*Grxcr2*^*tm1Dck*^) were intercrossed to generate three genotypic classes (+/+, *+/*Δ, and Δ*/*Δ). *Grxcr2* homozygote mutants (Δ*/*Δ) were also mated to FVB/NJ strain mice and progeny were sib mated to evaluate the effect of the *Grxcr2* deletion on this genetic background.

### Genotype analysis

A set of primers (5’-CATCAAGACCCCTGGAATTG-3’; 5’-ACAGCAACTGACACCCACAC-3’) designed from sequences 5’ of *Grxcr2* and flanking the proximal loxP site were used in standard PCR reactions to amplify genomic DNA from tail biopsies for identification of flox-neo allele status. These two primers, together with a primer derived from sequences in the neo cassette (5’-TAGTTGCCAGCCATCTGTTG-3’), were used in a multiplex PCR reaction for identification of the wild type (+) and the deleted (Δ) alleles after Cre-mediated recombination.

### RNA expression studies

Temporal bones were removed from mice ranging from P0 to adult time points and whole inner ear tissue was dissected in ice cold RNA Later (Ambion, Carlsbad, CA). Immediately prior to RNA extraction the RNA Later was removed and 1 mL of Trizol (Invitrogen; Carlsbad, CA) was added to each sample. The tissue was homogenized and RNA was extracted using the Purelink RNA Micro kit (Invitrogen; Carlsbad, CA). One μg of RNA was used as the input to a Super Script III reverse transcription reaction (Invitrogen; Carlsbad, CA) using oligo dT as a primer. One μL of the RT product along with *Grxcr2* gene-specific primers designed to exons 1 and 3 were used in a standard RT-PCR reaction. Separate reactions, also using 1 μL of the RT product, were performed in parallel to amplify the endogenous transferrin receptor (*Tfrc*) or *Gapdh* genes as internal controls. For quantitative RT-PCR (qRT-PCR) of *Grxcr2* expression during normal development, conditions for two sets of exonic primers, one set designed to exon 1 and exon 2 of *Grxcr2* and the other designed to the ribosomal subunit 16S gene, were optimized for specific product amplification and minimal primer dimer production. qRT-PCR reactions were performed in triplicate for three mice of each genotype and consisted of Sybr green master mix, 1 μL of the RT-product derived from whole inner ear RNA, and primers designed to *Grxcr2* exon 1 and exon 2 or the ribosomal subunit 16S. For qRT-PCR of *Grxcr2* expression in wild type and mutant cochleae from three month old mice, the same set of optimized *Grxcr2* exon 1 and exon 2 primers was used. Reaction cycling was performed in triplicate for each sample on an ABI 7300 Real-Time PCR system (Applied Biosystems; Foster City, CA) and gene expression was quantified using the ΔΔCt method [26]. Expression normalization was performed by computing average Ct values from gene-specific amplification of three control genes for each sample: *Trfc*; peptidylprolyl isomerase B (*Ppib*); and tubulin, beta 4B class IVB (*Tubb4b*). Fold-log expression means were compared by one-way ANOVA and Tukey’s post-hoc multiple comparison test using Graphpad Prism 7.0c.

### GRXCR2 antibody generation and immunostaining

A peptide designed from amino acids 68 to 83 of the primary sequence of mouse GRXCR2 (N-GEVPKPQPYSPKLTAQ-C) was covalently linked to KLH and injected into New Zealand rabbits (Proteintech Group; Chicago, IL). Antiserum was purified on an affinity column containing full-length recombinant GRXCR2 tagged at its N-terminus with maltose binding protein, using previously described methods [27]. Temporal bones were removed from mice at the appropriate age and fixed in 4% paraformaldehyde. The sensory epithelium was microdissected and the tectorial membrane was removed. Tissue was washed in PBS and permeabilized in 0.3% Triton-X for 10 minutes. Tissue was washed in PBS and incubated in 5% goat serum for 1 hour. A 1:900 dilution of phalloidin-Alexa 546 or phalloidin-rhodamine was applied to the tissue for 30 minutes and then the tissue was incubated overnight in a 1:100 dilution of the rabbit-derived, anti-GRXCR2 primary antiserum. Tissue was washed in PBS several times and incubated in a 1:300 dilution of goat anti-rabbit Alexa 488 secondary antibody. The tissue was again washed in PBS, mounted, and imaged on a Leica SPX5 2-photon laser-scanning confocal microscope. Images were processed using Photoshop CS2.

### Evaluation of auditory function

Mice were anesthetized with a combination of ketamine (65 mg/kg), xylazine (3.5 mg/kg), and acepromazine (2mg/kg). Body temperature was maintained with water circulating heating pads and heat lamps. Tucker Davis Technologies (TDT) System III hardware and SigGen/BioSig software (TDT, Alachua, FL USA) were used to present stimuli and record responses in an electrically and acoustically shielded chamber (Acoustic Systems, Austin, TX USA). For ABR measurements, sub-dermal needle electrodes were placed at the vertex (active), the test ear (reference) and the contralateral ear (ground). Stimulus tones were delivered through an EC1 driver (TDT, aluminum enclosure made in-house), with the speculum placed just inside the tragus. Stimulus presentations were 15 msec tone bursts, with 1 msec rise/fall times, presented at 10 per second. Up to 1024 responses were averaged for each stimulus level. Responses were collected for stimulus levels in 10 dB steps at higher stimulus levels, with additional 5 dB steps near threshold. Thresholds were interpolated between the lowest stimulus level at which a response was observed, and 5 dB lower, at which no response was observed. For DPOAE measurements, stimulus tones were delivered through two EC1 drivers (TDT, aluminum-shielded enclosure made in-house) connected with an Etymotic microphone (ER 10B+, Etymotic Research, Inc., Elk Grove Village, IL). The primary tones, F1 and F2, were set at a ratio of F2/F1 = 1.2. The intensity of F1 was varied in 5 or 10 dB steps, with the intensity of F1 ranging from 10–80 dB SPL and the intensity of F2 held at 10 dB lower than F1 intensity. DPOAE responses were measured at 2F1 –F2. Threshold means were compared using the nonparametic Mann-Whitney test with SSPS software (IBM). Endocochlear potentials were recorded as detailed in Raphael et al., 2001 [28]

### Evaluation of vestibular function

Mice were anesthetized with a mixture of ketamine (90–126 mg/kg) and xylazine (10–14 mg/kg). Body temperature was maintained with a homeothermic heating pad system (FHC, Inc.). Subdermal electrodes were placed just posterior to the lambdoidal suture (non-inverting), behind the right pinna (inverting), and at the hip (ground). TDT System II modules and custom software were used to present stimuli and record responses in an electrically shielded chamber using methods previously published [29]. Linear acceleration pulses (17 pulses/s, 2ms duration) were presented to the cranium in the naso-occipital axis. A noninvasive head clip was used to secure the head to a mechanical shaker for delivery of vestibular stimuli. Stimulus levels were quantified in decibels relative to 1.0 g/msec (1.0 g = 9.8 m/sec^2^) and ranged from +6 to -18 dB re: 1.0 g/msec adjusted in 3 dB steps. Electroencephalographic activity was amplified (200,000X), filtered (300–3000 Hz), and digitized (100 kHz). Two hundred fifty-six primary responses were averaged and replicated for each vestibular evoked potential (VsEP) waveform. The first positive and negative response peaks were scored for the VsEP waveforms as this initial response peak is generated by the peripheral vestibular nerve. Peak latencies (measured in milliseconds), peak-to-peak amplitude (measured in microvolts), and thresholds (measured in dB re: 1.0 g/msec) were quantified and mean values were compared across genotypes using analysis of variance (ANOVA). Post hoc comparisons were performed using least significant differences (LSD).

### Scanning electron microscopy

Temporal bones were removed from mice and placed in a fixative solution containing 2% glutaraldehyde and 0.2 M cacodylate buffer. The cochlea was microdissected to expose the sensory epithelium and incubated in 2% glutaraldehyde + 0.2 M cacodylate buffer for a minimum of 2 days. Tissue was washed in water and processed using the OTOTO method, which alternates the application of osmium tetroxide and thiocarbohydrazide [30]. The samples were critical point dried using a Samdri-790 critical point dryer (Tousimis Research Corporation; Rockville, MD) and mounted on stubs using colloidal silver paste. The samples were analyzed using an Amray 1000B scanning electron microscope. A minimum of five mice of each genotype and at each time point was evaluated.

### Quantification of bundle orientation

Sensory epithelia from the cochleae of *Grxcr2* heterozygous and homozygous mutant mice were dissected as whole mount preparations, fixed and immunostained with antibodies reactive with plastin/fimbrin isoforms [31] and with acetylated tubulin (Sigma Cat# T7451). Stereocilia bundle orientation was calculated using ImageJ software with laser scanning confocal images of the immunostained tissue. A line bisecting the midpoint of the bundle was drawn through the vertex, marked by the acetylated-α-tubulin signal. The angle between this line and a longitudinal reference line through pillar cells was measured. A total of 75 OHC bundles in *Grxcr2* heterozygotes and 75 OHC bundles in *Grxcr2* homozygotes were evaluated.

### FM1-43 imaging and electrophysiological recordings of hair cells

Sensory tissue was harvested from the cochleae and utricles of neonatal mice between postnatal days 0 to 6 (P0-P6). Tissue preparation, FM1-43 imaging and whole cell recordings of mechanically induced currents were performed as described previously [26,27]. Briefly, FM1-43 uptake was assessed with a differential interference contrast microscope equipped with a FM1-43 filter set within 5 min after 10 sec exposure to FM1-43 and 3 consecutive bath washes. Mechanoelectrical transduction currents were recorded at room temperature from acute preparations under whole cell patch clamp at a holding potential of -64mV. Recordings were performed in standard artificial perilymph solution containing (in mM): 144 NaCl, 0.7 NaH_2_PO_4_, 5.8 KCl, 1.3 CaCl_2_, 0.9 MgCl_2_, 5.6 D-glucose, and 10 HEPES-NaOH, adjusted to pH 7.4 and 320 mOsmol/kg. Vitamins (1:50) and amino acids (1:100) were added from concentrates (Invitrogen, Carlsbad, CA). Hair cells were viewed from the apical surface using an upright Axioskop FS microscope (Zeiss, Oberkochen, Germany) equipped with a 63X water immersion objective with differential interference contrast optics. Recording pipettes (2–5 MΩ) were pulled from borosilicate capillary glass (Garner Glass, Claremont, CA) and filled with intracellular solution containing (in mM): 135 KCl, 5 EGTA-KOH, 10 HEPES, 2.5 K_2_ATP, 3.5 MgCl_2_, 0.1 CaCl_2_, pH 7.4. Currents were recorded using the Axopatch Multiclamp 700A (Molecular devices, Palo Alto, CA), filtered at 10 kHz with a low pass Bessel filter, digitized at ≥20 kHz with a 12-bit acquisition board (Digidata 1322) and pClamp 8.2 (Molecular Devices, Palo Alto, CA) and stored on disk for offline analysis using OriginPro 7.1 (MicroCal Software, Northampton, MA). Hair bundles were deflected using a stiff probe mounted on a fast piezoelectrical bimorph with square step stimuli ranging from -0.2 μm to 1.15 μm. *Grxcr2* genotypes were determined post-analysis in a blinded fashion.

### Human variant screening

Variant screening was performed using genomic DNA from probands diagnosed with congenital, severe-to-profound, presumably nonsyndromic deafness or with Usher syndrome. In the majority of probands with nonsyndromic deafness, mutations in *GJB2* had been previously excluded. Probands with Usher syndrome had been previously screened with the Usher chip (Asper Biotech) for known mutations in 9 genes and/or by Sanger sequence analysis of a subset of the known Usher genes (*MYO7A*, *USH2A*, *CLRN1*). The three exons of *GRXCR2*, along with flanking non-coding sequences were amplified from genomic DNA of probands using standard PCR conditions (primer sequences available upon request) and purified products were analyzed by Sanger sequencing as previously described [8]. DNA sequences obtained from probands were compared to Genome Reference Consortium Human Build 37 (GRCh37; hg19) and to a reference *GRXCR2* cDNA sequence (NM_001080516.1). Allele frequencies of identified sequence variants were obtained from data available on the web from the 1000 Genomes Project [32] and NHLBI Exome Sequencing Project. dbNSFP v2.0 was used to estimate evolutionary conservation of nonsynonymous variants and their potential effects on protein structure [33]. This database queries predictions and annotations from two conservation tools (GERP++ and PhyloP) and four prediction tools (SIFT, Polyphen2, LRT and MutationTaster).

## Results

### *Grxcr2* encodes a vertebrate-specific cysteine-rich protein expressed in the inner ear

**Fig 1:**
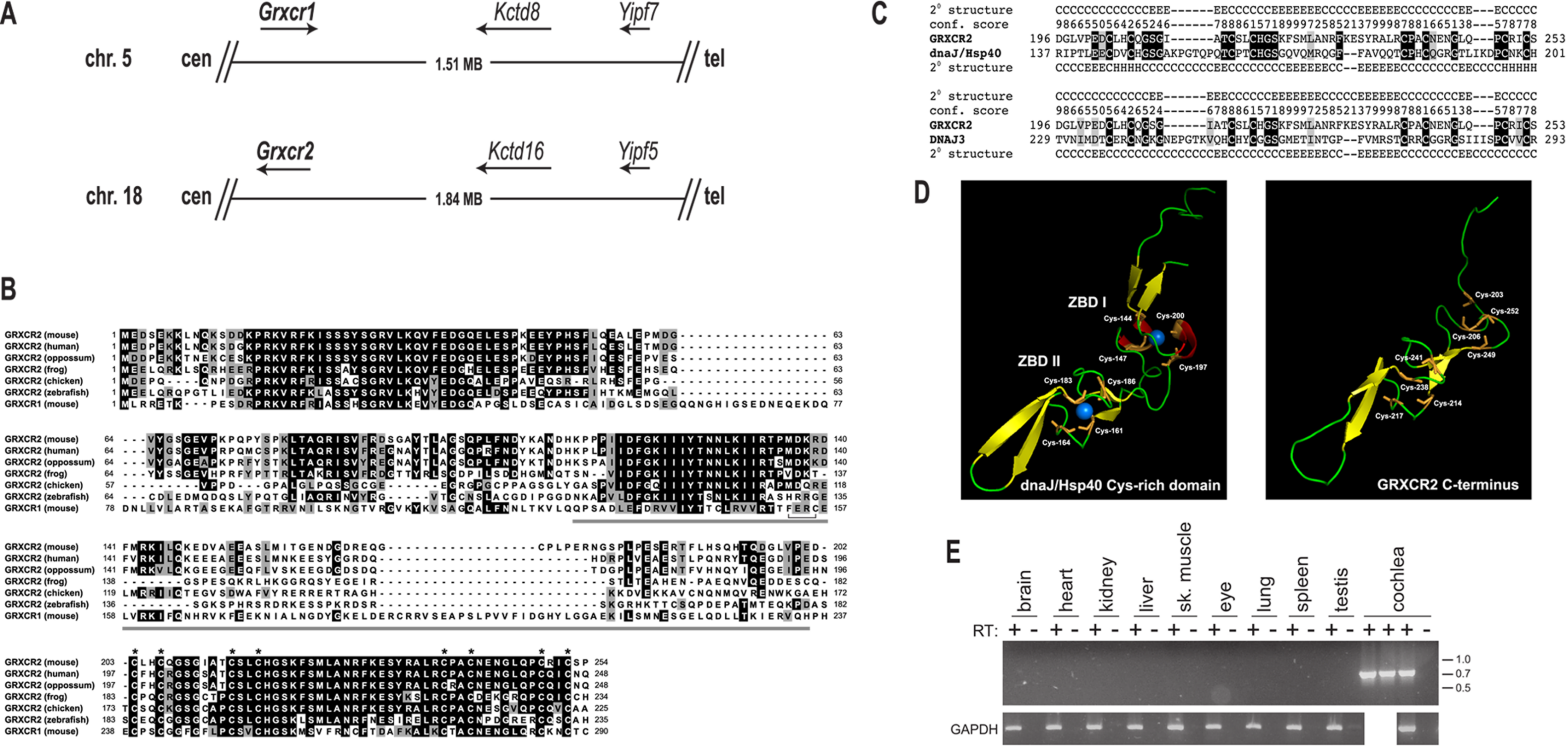
*Grxcr2* is paralogous to the previously identified deafness-associated gene *Grxcr1*. (A) Regions of mouse chromosomes 5 and 18 that contain the *Grxcr1* and *Grxcr2*, respectively, also contain additional conserved gene pairs (*Kctd8* and *Kctd16*; *Yipf7* and *Yipf5*) that are indicative of an ancestral duplication. Cen, centromere; tel, telomere. (B) Amino acid sequence comparison of GRXCR2 orthologs in a range of vertebrate species and of mouse GRXCR1 indicates strong conservation of the N- and C- termini, while the central domains are more divergent. Gray bar, thioredoxin-like domain of GRXCR1; bracket, putative GRXCR1 active site. Asterisks, conserved bipartite arrangement of cysteine residues. Black or gray shading indicates positions at which residues from at least four species are identical or biochemically similar, respectively. (C) Predicted secondary structure of the C-terminal Cys-rich domain of GRXCR2 exhibits significant similarity to Cys-rich domains present in *E*. *coli* dnaJ/Hsp40 (P08622) and human DNAJ3 (NP_005138) proteins. PSI-BLAST alignments are shown along with secondary structural information (H, alpha helix; E, beta strand; and C, coil) and associated confidence scores for the GRXCR2 predictions derived from analysis with Phyre^2^ (9, highest; 0, lowest). E values supporting significance of the PSI-BLAST alignments (three iterations) were 4x10^-10^ (dnaJ/Hsp40) and 2x10^-8^ (DNAJ3). (D) Structure of the dnaJ/Hsp40 Cys-rich domain bound to two zinc ions is similar to the top scoring model of the GRXCR2 C-terminal domain predicted by I-TASSER using the MUSTER threading algorithm [36]. The confidence score supporting this GRXCR2 model was -0.41, consistent with an RMSD of 3.7 ± 2.5 angstroms with respect to dnaJ/Hsp40. Secondary structure is indicated by color: red, alpha helix; yellow, beta strand; and green, coil. Zinc ions are indicated in blue and Cys residues comprising zinc binding domain I (ZBDI) and ZBDII are indicated in orange. (E) RT-PCR products corresponding to full-length *Grxcr2* transcripts were amplified from cochlear RNA, but not from RNA derived from a variety of other adult tissues. Molecular weight markers are indicated at right, in kilobases. RT, reverse transcriptase.

*Grxcr2* is located on mouse chromosome 18 in a region paralogous with the *Grxcr1* locus (Fig 1A). Two additional conserved gene pairs, *Kctd8*/*Kctd16* and *Yipf7*/*Yipf5*, lie within 1 Mb of *Grxcr1* and *Grxcr2* on mouse chromosomes 5 and 18, respectively, consistent with derivation of these regions from a larger ancestral duplication. The presence of *Grxcr1* and *Grxcr2* orthologs in extant vertebrates but only a single related gene in most non-vertebrate species (S1 Fig) suggests that the duplication event that gave rise to these two genes occurred early in vertebrate evolution. Further analysis of the corresponding syntenic regions on human chromosomes 4 (*GRXCR1*) and 5 (*GRXCR2*) indicates that a much larger set of genes (approximately 120) are part of these paralogous clusters [34] and is consistent with models of genome-wide duplication events [35].

*Grxcr2* encodes a predicted protein of 254 amino acids with approximately 30% amino acid identity with GRXCR1, with complete conservation of the arrangement of cysteines (CX_2_CX_7_CX_2_CX_20_CX_2_CX_7_C) in the C-terminal region. (Fig 1B). The central region of GRXCR1 exhibits similarity in primary sequence and predicted secondary structure to glutaredoxin proteins, members of the large thioredoxin family of proteins [7]. Thioredoxin-related proteins typically possess oxidoreductase activity and catalyze thiol exchange reactions [37]. The central domains of GRXCR1 and GRXCR2 retain low-level identity (20%) with one another. Key residues in glutaredoxins, including putative active site cysteines, however are absent in GRXCR2, indicating that GRXCR2 is unlikely to possess intrinsic oxidoreductase activity. *Grxcr2* orthologs are present in vertebrate species including human, opossum, chicken, frog, and fish with the most conserved residues at the N- and C-termini, including complete conservation of cysteine residues of the C-terminal domain (Fig 1B). The more divergent, central region of GRXCR2 varies in relative size among orthologs and with GRXCR1.

The arrangement of cysteine residues in the C-terminal region of GRXCR2 is similar to that found in the Cys-rich domains present in dnaJ-related proteins. This family of proteins act as co-chaperones and provide functional specificity for heat shock protein 70 (Hsp70) to control protein conformation in a variety of contexts, including proper folding of newly translated proteins, refolding of proteins after cellular stress, and modulation of protein-protein interactions [38]. We identified significant similarity in the predicted structure of this region of GRXCR2 and the known secondary structures of several dnaJ-related proteins, including *E*. *coli* dnaJ/Hsp40 and human DNAJ3 (Fig 1C). The Cys-rich domain of dnaJ/Hsp40 coordinates interactions with two zinc ions and is required for optimal binding of native and misfolded substrate proteins by the Hsp70 complex [39, 40]. A predicted structural model of the C-terminal domain of GRXCR2 suggests an arrangement of Cys residues into two clusters that are similar to the two zinc binding domains (ZBD) demonstrated in dnaJ/Hsp40, particularly in the region corresponding to ZBD II (Fig 1D).

*Grxcr2* transcripts containing the three coding exons of the gene were observed in RNA prepared from the cochleae of wild type mice at 3 weeks of age but were undetectable in RNA from a variety of other tissues (Fig 1E). Expression of Grxcr2 has been demonstrated in other tissues (e.g., brain and pituitary gland [41, 42]) but only at low levels, consistent with relatively selective expression of the gene in the inner ear.

### Targeting the *Grxcr2* locus to derive *Grxcr2* mutant mice

**Fig 2:**
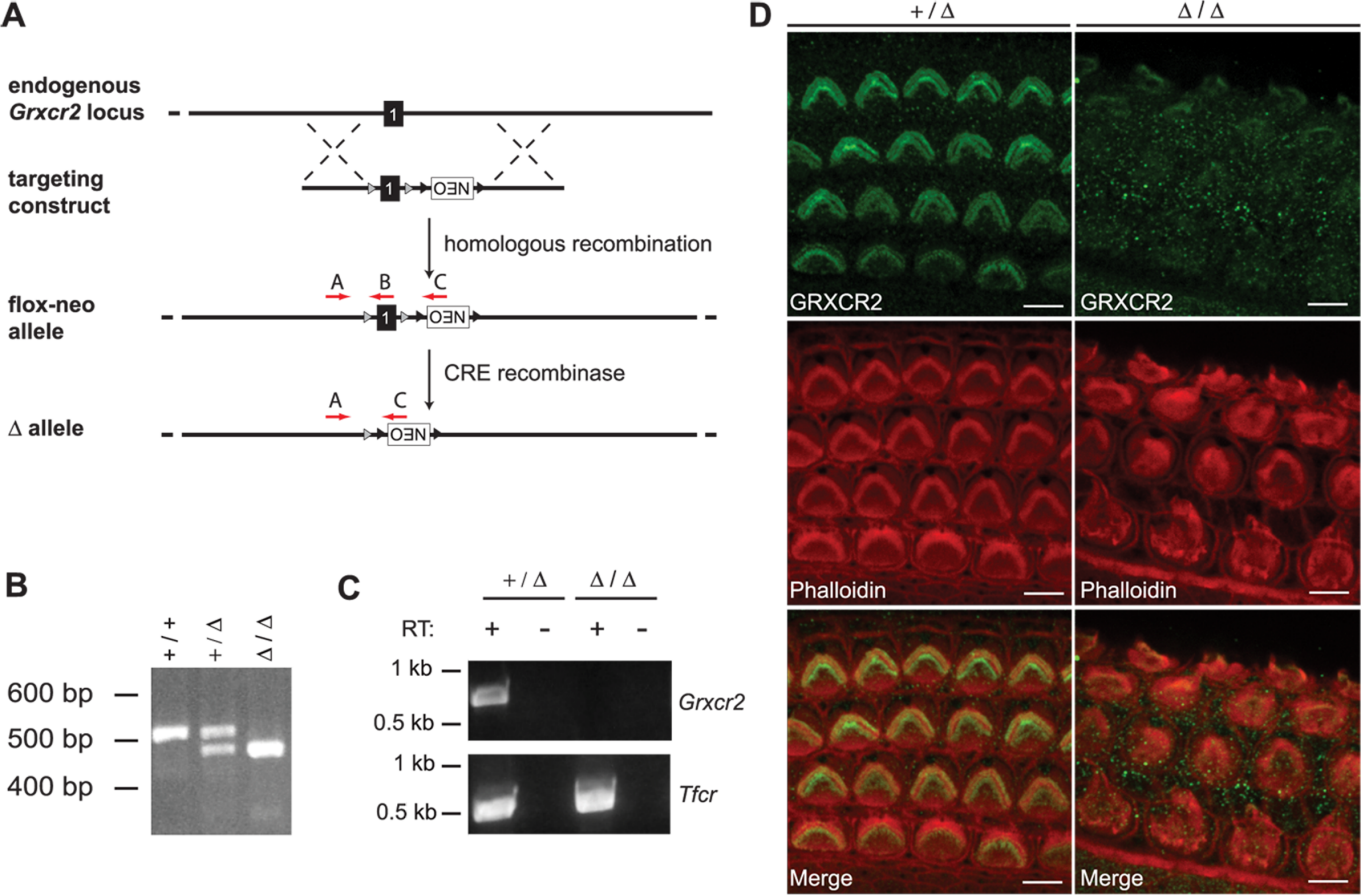
Mutational targeting of exon 1 of *Grxcr2*. (A) The wild type *Grxcr2* locus, the recombinant flox-neo allele after homologous integration of the targeting construct, and the deleted (Δ) allele after Cre recombination are shown. Red arrows indicate the positions of genotyping primers to detect the endogenous (primer pair A and B) and deleted (primer pair A & C) alleles of *Grxcr2*. (B) Triplex PCR of genomic DNA using primers A, B, and C generated a 532 bp product from wild type mice (*+/+*), a 490 bp product from homozygous mutants (Δ/Δ), and both products from heterozygotes (*+/*Δ). (C) RT-PCR of cochlear RNA from heterozygous *+/*Δ mice generated a 719 bp product, indicating expression of normal *Grxcr2* transcripts. The absence of this product from Δ/Δ mutants demonstrated loss of normal *Grxcr2* expression. Amplification of *Tfrc* transcripts from cochlear RNA of both genotypes demonstrated similar RNA quantity and quality. *Tfrc*, transferrin receptor; RT, reverse transcriptase. (D) GRXCR2 immunoreactivity was demonstrated in stereocilia bundles in the cochlea of Grxcr2 heterozygotes at postnatal day 3 but was absent from Δ/Δ mutants. Phalloidin reactivity indicates actin filament content in the stereocilia of mice of both genotypes. Scale bars indicate 5 μm.

Based upon its conservation in sequence and expression pattern with *Grxcr1*, we hypothesized a similar requirement for *Grxcr2* in normal development and function of the inner ear. To directly test this, we generated a targeted deletion of exon 1 of *Grxcr2* using homologous recombination in mouse ES cells and the strategy summarized in Fig 2A. Intercross of mice carrying germline deletions of *Grxcr2* (+/Δ) produced offspring at weaning age of the three expected genotypes (Fig 2B) consistent with expected Mendelian ratios (*N* = 46; χ^2^ = 2.13, *p* = 0.34), indicating that *Grxcr2* is not critical for general viability. RT-PCR analysis of cochlear RNA demonstrated that heterozygote carriers of the deleted *Grxcr2* allele (+/Δ) expressed full-length *Grxcr2* transcripts (Fig 2C). Full-length transcripts were absent in mice homozygous for the *Grxcr2* mutant allele (Δ/Δ), consistent with deletion of exon 1 (Fig 2C). Using antiserum generated against a GRXCR2 peptide encoded by exon 1, we detected immunoreactivity in the stereocilia of heterozygous animals (Fig 2D). This reactivity was diminished to nonspecific background levels in cochlear tissue from homozygous mutants, consistent with loss of exon 1 and ablation of GRXCR2 expression.

### *Grxcr2* mutants exhibit significant hearing loss

**Fig 3:**
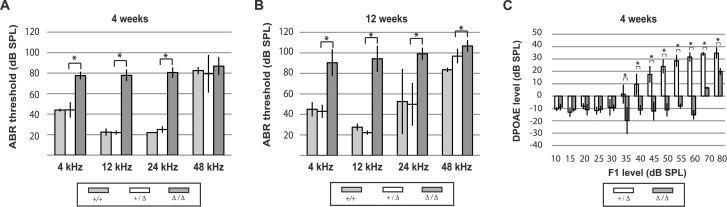
*Grxcr2* mutants exhibit auditory defects including outer hair cell dysfunction. (A) At 4 weeks of age, *Grxcr2* homozygous mutants (n = 7) exhibited increased ABR thresholds in response to pure tones at 4, 12, and 24 kHz relative to wild type (n = 2) and heterozygous (n = 7) littermates (p < 0.05). Vertical bars here and in (B) and (C) represent standard deviations. Asterisks represent probabilities of statistically significant differences in threshold means (*, p < 0.05). (B) Similar ABR threshold increases were demonstrated in *Grxcr2* homozygous mutants (n = 7) relative to wild type (n = 2) and heterozygous (n = 6) littermates at 12 weeks of age (p < 0.05). (C) At 4 weeks of age, *Grxcr2* homozygotes (n = 5) exhibited significantly reduced DPOAE in response to 12 kHz stimuli, relative to DPOAE of heterozygous littermates (n = 5) (p < 0.05).

*Grxcr2* Δ/Δ mice responded to loud sound with startle reflexes at two weeks of age, the time of onset of hearing in normal mice, indicating at least some auditory function in the mutants. Endocochlear potentials, a resting potential present in the cochlea that is required for hearing [43], were normal in the Δ/Δ mice at three months of age (Δ/Δ, 107 and 100 mV (N = 2); +/Δ, 98 mV (N = 2)). For a more objective assay of hearing ability, we evaluated auditory brainstem responses (ABR), a measure of synchronous auditory activity in response to sound, at a range of stimulus frequencies at 4 and 12 weeks of age. Wild type (+/+) and *Grxcr2* heterozygote (+/Δ) animals exhibited relatively low ABR thresholds in response to 4, 12, and 24 kHz tones at 4 weeks of age (Fig 3A). In contrast, hearing thresholds at these frequencies in *Grxcr2* Δ/Δ mice were elevated by approximately 30 to 60 dB above those of both +/+ and +/Δ mice, indicating recessive inheritance of a hearing loss phenotype (Fig 3A). Similar ABR threshold shifts in response to 4, 12, and 24 kHz tones were observed at 4 weeks of age in homozygous mutants from a second *Grxcr2* line that was derived from an independently targeted ES clone, verifying the role of mutant *Grxcr2* in the abnormal phenotype (S2 Fig). ABR measurements at 12 weeks of age indicated an average threshold shift of 40 to 70 dB in response to 4,12, and 24 kHz tones in *Grxcr2* Δ/Δ mice relative to littermate controls (Fig 3B). The 10 to 20 dB progression in hearing loss between 4 and 12 weeks in Δ/Δ mice was statistically significant (p < 0.01) for 12, 24, and 48 kHz stimuli but not for 4 kHz. High thresholds in response to 48 kHz tones were observed in mice of all three genotypes and likely reflected the effects of the *Cdh23* allele in the C57BL/6 background, which predisposes many inbred strains to age-related hearing loss (ARHL) that typically initiates at high frequencies [44–46]. Consistent with this, mice carrying the Δ/Δ mutation on a C57BL/6 x FVB/NJ F2 hybrid background with ARHL-resistant alleles of the *Cdh23* gene exhibited 40 to 70 dB threshold shifts at all tested frequencies, including 48 kHz, relative to +/+ controls (S3 Fig) and indicating that hearing loss in the *Grxcr2* mutant is independent of *Ahl* allele status.

We evaluated distortion product otoacoustic emissions (DPOAE) as an additional measurement of hearing ability of *Grxcr2* mutants. DPOAE are responses of the cochlea that are evoked by delivery of 2 tones of similar frequency to the ear and are principally due to the amplification activity of outer hair cells [47]. At 4 weeks of age, littermate +/Δ mice exhibited a stepwise increase in DPOAE in response to 12 kHz stimuli greater than 30 dB in intensity (Fig 3C). In contrast, *Grxcr2* Δ/Δ mutants exhibited significant DPOAEs only in response to stimuli at 70 and 80 dB SPL (Fig 3C). These responses to very high intensity stimuli likely represent distortion artifacts that are present even in euthanized animals [48]. Similar reduced emissions in response to stimuli at 24 kHz were observed in *Grxcr2* mutants, indicating outer hair cell dysfunction at other locations along the cochlea (S4 Fig).

### Hearing loss in *Grxcr2* mutants is associated with defects in stereocilia orientation and organization

**Fig 4:**
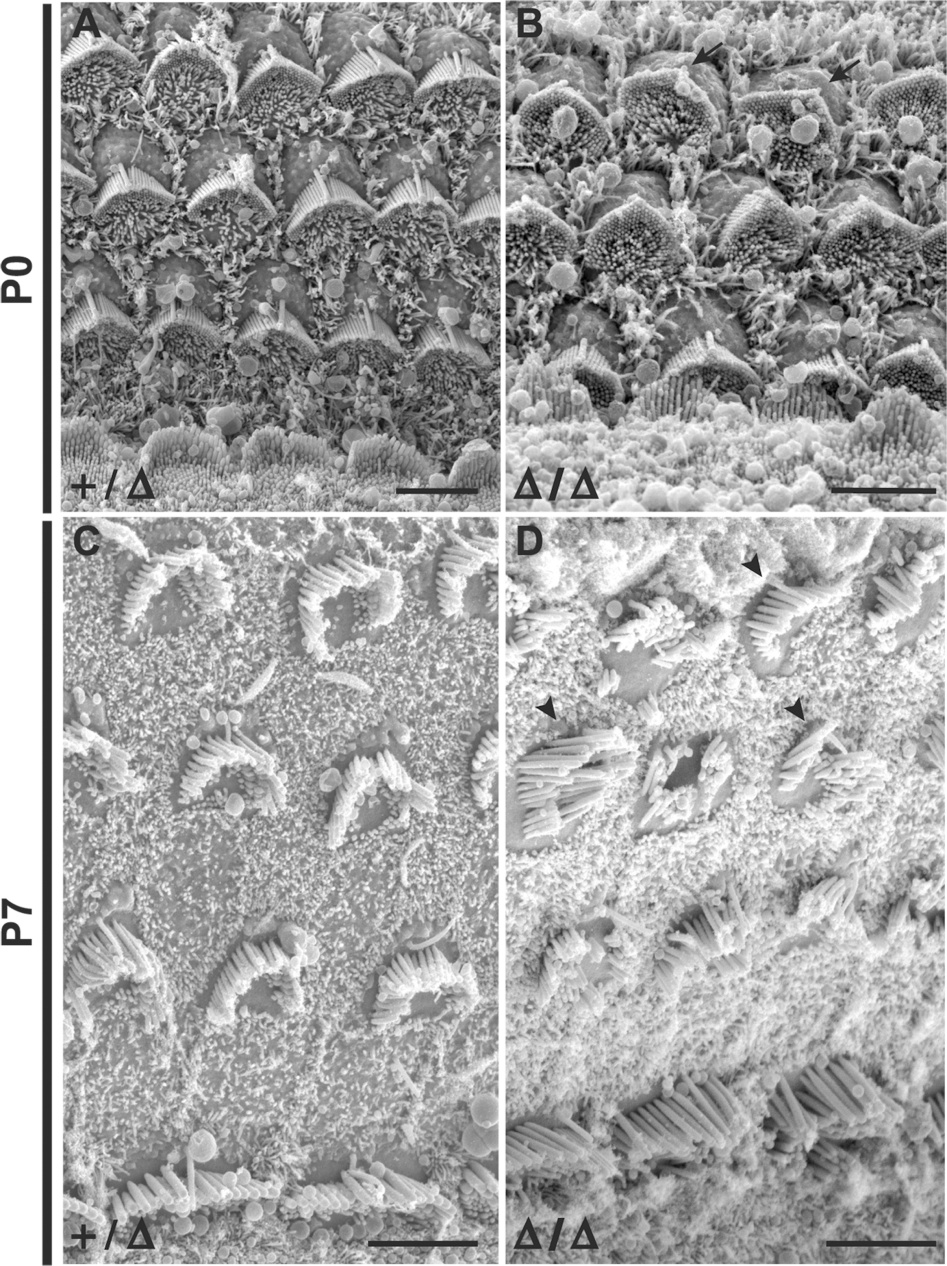
*Grxcr2* mutant mice exhibit stereocilia defects at early postnatal ages. (A) and (C) Scanning electron micrographs of the cochlear sensory epithelium from *Grxcr2* heterozygotes demonstrated an organized mosaic of inner and outer hair cells and normal stereocilia bundle morphology at P0 and P7. (B) The cochlear sensory epithelium from *Grxcr2* mutant homozygotes exhibited normal organization of sensory cells. Bundle morphology was also relatively normal, but bundles on outer hair cells exhibited slight deviations in orientation at P0. (D) At P7, outer hair cell bundles of *Grxcr2* homozygotes exhibited more severe orientation defects and were severely disorganized, including flattened, splayed, and distorted bundles (arrowheads). All scale bars indicate 5 μm. All images are taken from positions in the cochlea approximately one-half turn from the apex. Similar bundle defects were observed in more basal regions of the mutant cochleae.

In order to identify a potential developmental correlate of the hearing loss observed in *Grxcr2* mutant mice, we used scanning electron microscopy (SEM) to evaluate cochlear morphology at early postnatal time points. During the first postnatal week, *Grxcr2* heterozygotes exhibited a regular arrangement of the sensory epithelium, including a single row of inner hair cells and three rows of outer hair cells along the length of the cochlea (Fig 4A and 4C). The apical surface of the sensory cells contained well-organized, ‘V’ shaped stereocilia bundles with a single kinocilium at their vertices and orientated with axes perpendicular to the longitudinal axis of the cochlea (Fig 4A and 4C). Overall organization of inner and outer hair cells was also normal in cochleae of *Grxcr2* Δ/Δ mice (Fig 4B and 4D). At P0, however, many stereocilia bundles in Δ/Δ mutants exhibited subtle deviations in their orientation (Fig 4B). These polarity defects were more obvious in bundles on outer hair cells. One week later (P7), progression of stereocilia defects was observed in Δ/Δ mutants (Fig 4B and 4D). Most bundles on outer hair cells in the mutants exhibited orientation defects, with many showing more exaggerated deviation from the normal axis of polarity. In addition, most bundles were lacking the normal “V” shape found in control mice. Extreme disorganization was often observed, including examples of split, splayed, and severely distorted bundles (Fig 4D, arrowheads). Bundle disorganization was also noted on some inner hair cells (Fig 4D; also Fig 2D).

**Fig 5:**
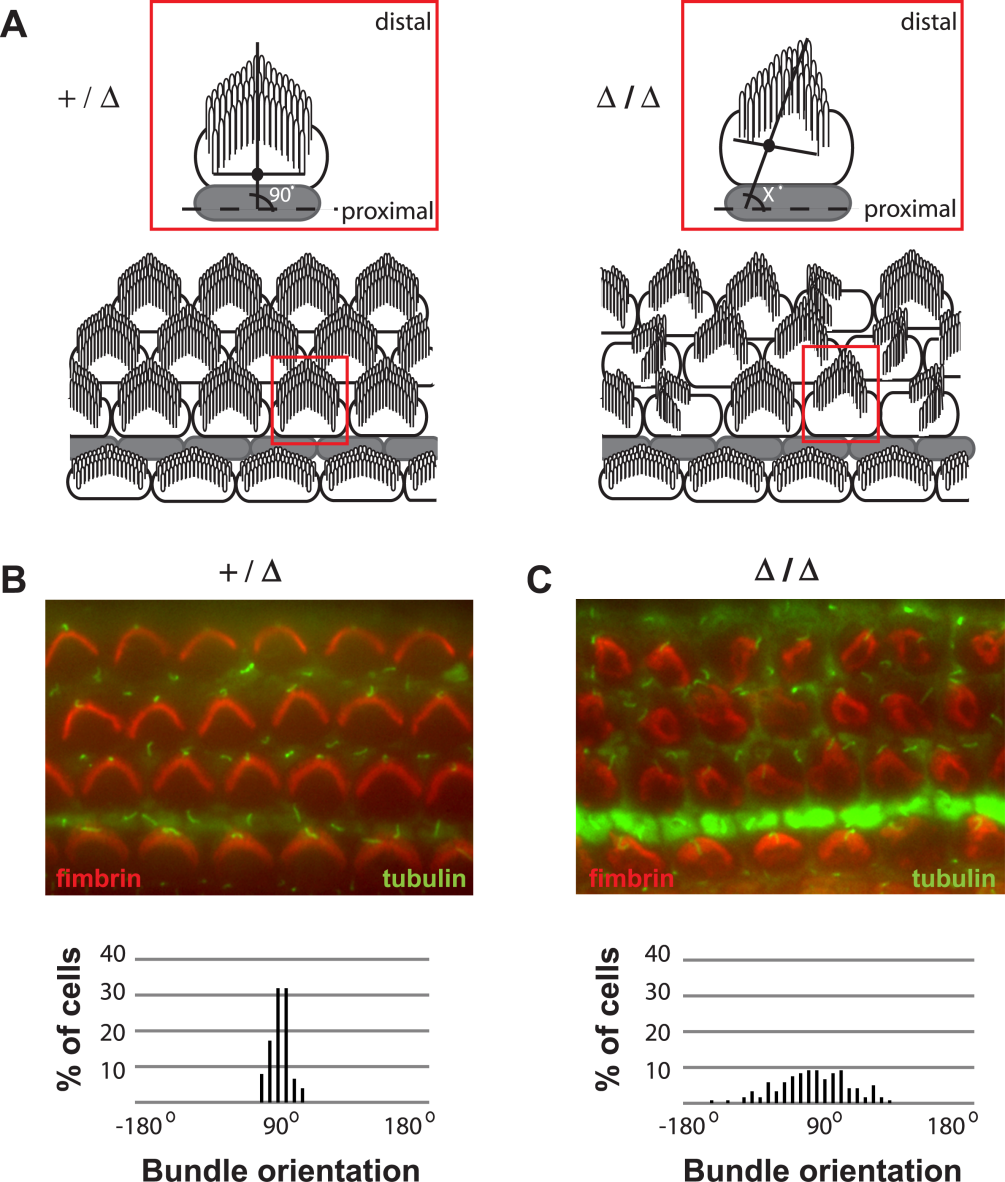
Loss of *Grxcr2* function results in stereocilia orientation defects. **(**A) Schematic representations of the orientation of stereocilia bundles in *Grxcr2* heterozygotes and homozygous mutants are shown. Bundle orientation was calculated by identifying the kinocilium at the vertex of the bundle and the mid-point (solid black dot). The angle between a line extended through these two landmarks and a longitudinal reference line through pillar cells was measured. (B) Cochlea from a *Grxcr2* heterozygote at P0 was stained with phalloidin (red) to mark the stereocilia and acetylated tubulin (green) to mark the kinocilium. The bundles appeared morphologically normal with a kinocilium at the vertex of each bundle. The angles measured show a tight distribution around 90 degrees with respect to the longitudinal axis of the cochlea. (C) Kinocilia were present at the vertex of bundles from the cochlea of a *Grxcr2* homozygote at P0, but bundles were misoriented with significant deviation from 90 degrees.

We further characterized the early postnatal defects in stereocilia bundles by treating cochleae dissected at P0 from *Grxcr2* Δ/Δ and +/Δ mice with antibodies specific for fimbrin/plastins, actin bundling proteins present in the core of stereocilia [31] and for acetylated tubulin, which immunostains the microtubules present in the kinocilia of developing hair cells. All bundles in *Grxcr2* +/Δ cochleae exhibited tubulin-positive kinocilia at their vertices and orientations of the bundles axes were tightly distributed around 90^0^ with respect to the longitudinal axis of the cochlea (Fig 5B), consistent with SEM analyses (Fig 4). In *Grxcr2* Δ/Δ cochleae at this age, tubulin-positive kinocilia were also observed at the vertices of bundles. However, the orientation of bundle axes exhibited significantly greater deviation from 90^0^ and suggests a defect in control of bundle polarity in the Δ/Δ mutants (Fig 5C). The extent of hearing loss in *Grxcr2* mutants, together with the severe bundle defects observed on outer hair cells, is consistent with loss of the normal amplification function of these sensory cells [49].

### Mechanotransduction is acquired normally in mice lacking *Grxcr2*

**Fig 6:**
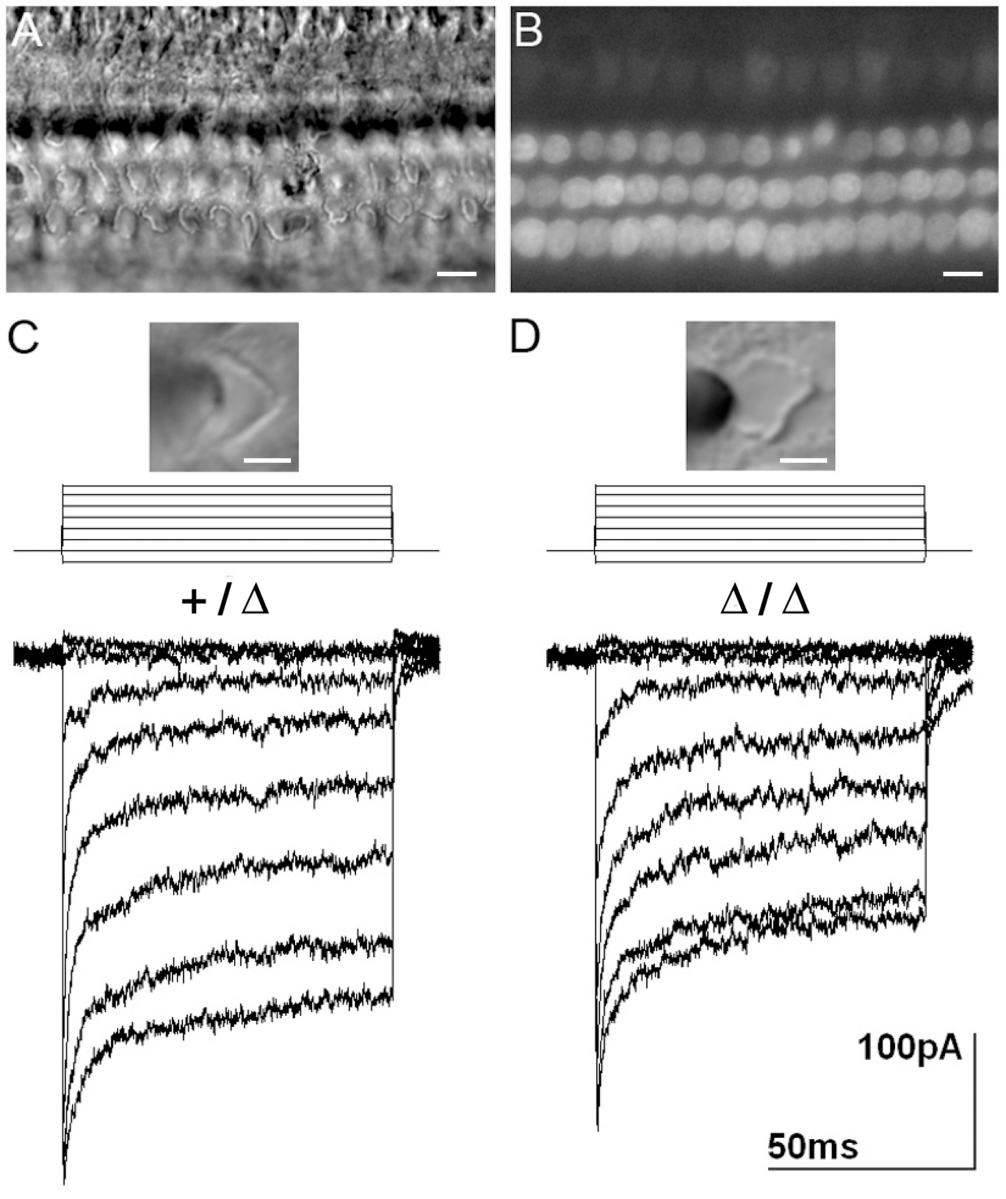
Mechanosensitivity is preserved in neonatal hair cells of homozygous *Grxcr2* mutant mice. **(**A) Differential Interference contrast (DIC) images of the mid-apical turn at P6 shows disorganized hair bundles in Δ/Δ mutants. (B) FM1-43 uptake into hair cells in the same section appears normal, suggesting functional mechanotransduction channels are present. (C) and (D) Comparable transduction currents are evoked in mid-basal outer hair cells of heterozygous mice (C) and homozygous mutants (D) at P2. Bundle displacements ranged from -0.2 to 1.2 μm. Corresponding DIC images depict hair bundles before application of the stimulus pipette. Scale bars indicate 10 μm (A and B) and 3 μm (C and D).

To assess whether mechanotransduction develops normally in mice lacking *Grxcr2*, we analyzed uptake of the styryl dye FM1-43 in sensory hair cells of neonatal *Grxcr2* Δ/Δ and +/Δ mice. Brief applications (10 sec) of FM1-43 (5μM) are routinely used to assess the presence of functional mechanosensitive channels open at rest in hair cells of the inner ear [50–53]. Despite the presence of misoriented and disorganized bundles in the Δ/Δ mice (Fig 6A), FM1-43 uptake was comparable in Δ/Δ and +/Δ mice from P1 to P6 and similar to that described previously in the developing organ of Corti of wild type mice [51]. FM1-43 uptake was observed in sensory hair cells at the base of the organ of Δ/Δ mutants at P1 and the uptake progressed into hair cells in the apex by P6, the latest developmental stage analyzed (Fig 6B). To confirm the presence of functional transduction channels in stereocilia, outer hair cells were placed under whole cell voltage clamp configuration and currents induced by small displacements of the hair bundles were recorded at a physiologically relevant holding potential of -64mV. Similar transduction currents were evoked in both +/Δ and Δ/Δ mice (Fig 6C and 6D). The maximal transduction current amplitude evoked in outer hair cells was similar for both genotypes: 403.3 ± 63.1pA (n = 3, P2 and n = 1, P4) for +/Δ mice and 393.2 ± 46.4 pA (n = 3, P2 and n = 1, P1+1day in culture) for Δ/Δ mutants. Similar time constants were also demonstrated for the fast and slow components of adaptation of the transduction responses (*Grxcr2* +/Δ: Tau fast = 0.74 ± 0.20ms, Tau slow = 10.11 ± 7.97ms; *Grxcr2* Δ/Δ: Tau fast = 1.57 ± 0.88ms, Tau slow = 10.35 ± 6.13ms). A larger 10–90% operating range was observed in Δ/Δ mutants at 1.44 ± 0.47 μm compared to 0.97 ± 0.10 μm in +/Δ mice, which may reflect more subtle alterations of the transduction machinery in the disorganized bundles of the mutants. The amplitudes, kinetics, and operating ranges of transduction currents in the +/Δ and Δ/Δ mice were comparable to those described in wild type mice [51, 54].

### *Grxcr2* mutants exhibit vestibular dysfunction

**Fig 7:**
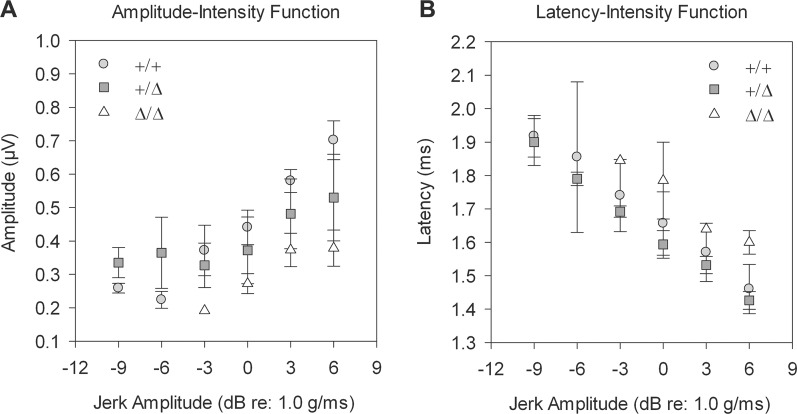
*Grxcr2* mutants exhibit altered responses to linear acceleration. (A) At 5 months of age, P1-N1 response amplitudes of /Δ mutants (n = 4) were decreased relative to +/Δ (n = 5) and +/+ (n = 3) mice, consistent with significantly elevated thresholds observed in the Δ/Δ mutants (ANOVA, F(2,11) = 11.442, p = 0.003). Post hoc analysis revealed that Δ/Δ mutant thresholds were significantly higher than +/Δ (p = 0.004) and +/+ (p = 0.002) mice. Thresholds for +/Δ and +/+ mice were statistically equivalent. (B) P1 response latencies of Δ/Δ mutants were increased at +6 dB (ANOVA, F(2,11) = 5.23, p = 0.03), indicating altered response timing in the mutants. Post hoc analysis revealed that latencies for Δ/Δ mutants were significantly longer than +/Δ mice (p = 0.012).

*Grxcr2* Δ/Δ mice do not exhibit circling or head shaking behaviors that are indicative of vestibular defects and that are often observed in mouse deafness mutants [55]. In addition, *Grxcr2* Δ/Δ mice have swimming abilities like those of +/Δ and +/+ littermates. This lack of overt behavioral defects in the mutants is consistent with the relatively normal bundle morphology observed in vestibular organs (S5 Fig). In addition, we have demonstrated transduction currents in vestibular hair cells of early postnatal Δ/Δ mutants that are consistent with normal sensory function (S6 Fig). To determine whether the mutants may have subtler vestibular dysfunction, we analyzed vestibular evoked potentials (VsEPs) in response to transient linear accelerations. These compound action potentials reflect activities of the eighth nerve and central vestibular neurons that depend upon sensory function of the utricle and saccule organs in the vestibular portion of the inner ear [56, 57]. P1-N1 response amplitudes of Δ/Δ mutants were decreased relative to +/Δ and +/+ mice at stimulus levels that elicited a measurable response, consistent with the significantly elevated thresholds observed in the Δ/Δ mutants (Δ/Δ, 0.75 ± 4.5 dB re: 1.0g/msec; +/Δ, -8.5 ± 2.5 dB; and +/+, -10.5 ± 3 dB) and indicating decreased sensitivity of the vestibular gravity receptor system (Fig 7A). The temporal characteristics of peripheral nerve activation were also altered. P1 response latencies of Δ/Δ mice were prolonged relative to +/Δ and +/+ mice at stimulus levels that elicited a measurable response (Fig 7B).

### Expression of *Grxcr2* in the inner ear

**Fig 8:**
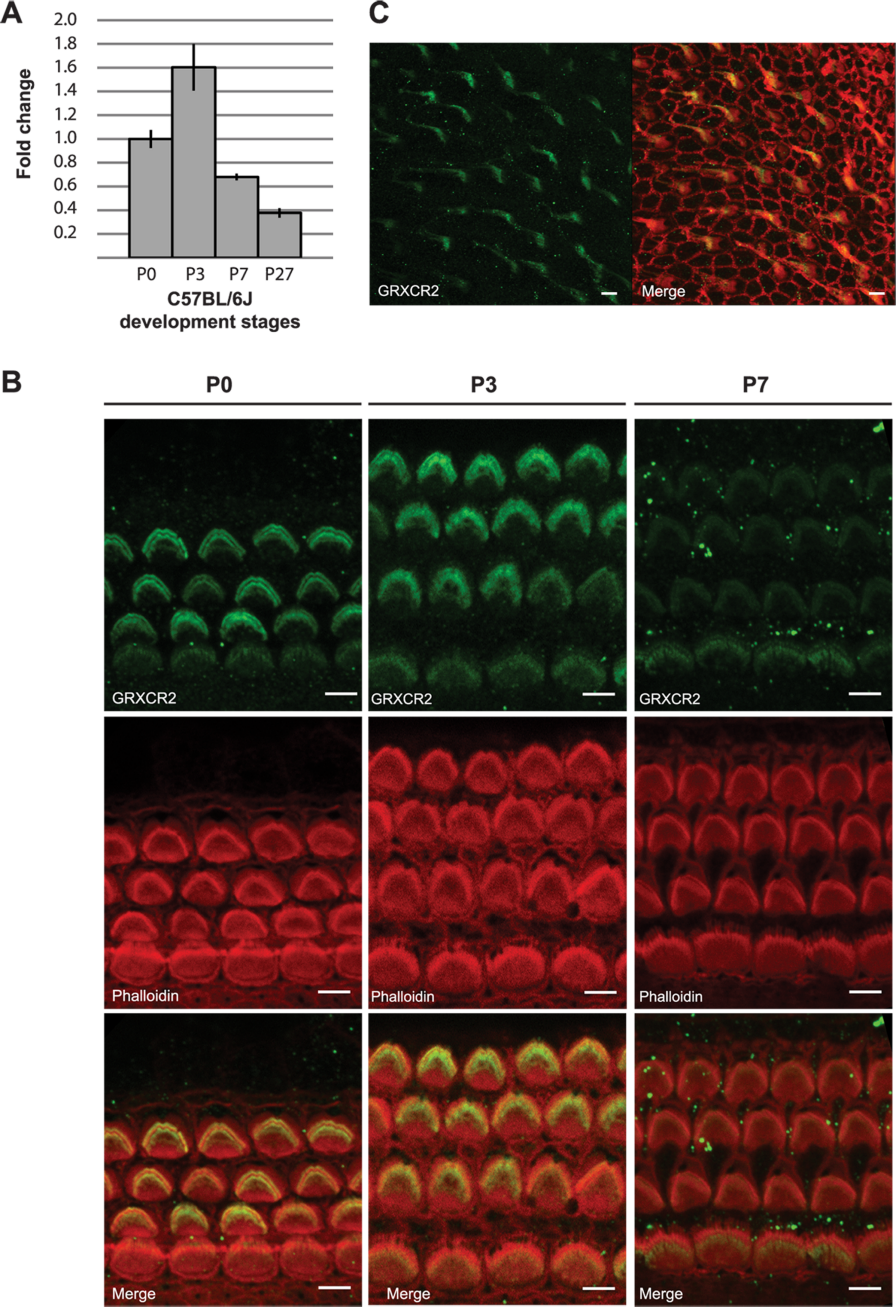
*Grxcr2* transcript and protein exhibit peak levels in the cochlea at early postnatal time points. (A) *Grxcr2* transcript levels in the cochleae of C57BL/6J mice at early postnatal time points and in adults were determined by qRT-PCR and normalized to those present at birth (P0). *Grxcr2* expression peaked at P3 and declined through the first postnatal week and young adulthood (P27). Vertical bars represent standard deviations. (B) The sensory epithelium from the cochleae of C57BL/6J mice was immunostained using an anti-GRXCR2 antibody and phalloidin to mark the stereocilia bundles. Outer hair cell bundles of mice at P0 and P3 exhibited robust GRXCR2 reactivity while bundles from P7 mice exhibited lower reactivity. Images are derived from the middle regions of the cochlea. Scale bars indicate 5 μm. (C) Stereocilia of sensory hair cells in the utricle at P0 were also reactive with the anti-GRXCR2 antibody (left, anti-GRXCR2; right panel, anti-GRXCR2 signal merged with phalloidin-rhodamine signal). Scale bars indicate 5 μm.

Quantitative RT-PCR (qRT-PCR) using *Grxcr2* specific primers was performed on RNA prepared from whole inner ears of C57BL/6J mice during the first postnatal week and at 1 month of age after the cochlea is mature. *Grxcr2* transcript levels were normalized to those present at birth in order to survey the transcript profile over time. *Grxcr2* RNA expression exhibited a relative increase from birth to P3 and declined through the first postnatal week and into maturity (Fig 8A). To evaluate expression of GRXCR2, the sensory epithelium was dissected from C57BL/6J mice and immunostained using the antiserum raised against a peptide derived from the N-terminal region of GRXCR2. The specificity of this antiserum is supported by the loss of reactivity in the stereocilia of *Grxcr2* Δ/Δ mutants (Fig 2D). In the mid-cochlear region at birth, the primary site of GRXCR2 immunoreactivity was in stereocilia of inner and outer hair cells, with higher levels of protein in outer hair cells (Fig 8B). Expression persisted in inner and outer hair cell stereocilia at postnatal day 3 and appeared reduced in outer hair cells at 1 week after birth, consistent with the decreased level of *Grxcr2* transcripts at this time (Fig 8B). A similar time course of stereocilia expression was also observed at other locations along the length of the cochlea (S7 Fig). We also observed localization of GRXCR2 in the stereocilia of vestibular organs (Fig 8C). We did not detect GRXCR2 in other cell types in the cochlea, including auditory neuron (spiral ganglion) cells. Enrichment of *Grxcr2* transcripts in hair cells, as well as the early postnatal peak of expression, were also demonstrated in published RNA-Seq studies using purified cell populations from the cochlea and the vestibular portions (utricle) of the inner ear [58, 59].

### Progressive hearing loss in a *Grxcr2* hypomorphic mutant

**Fig 9:**
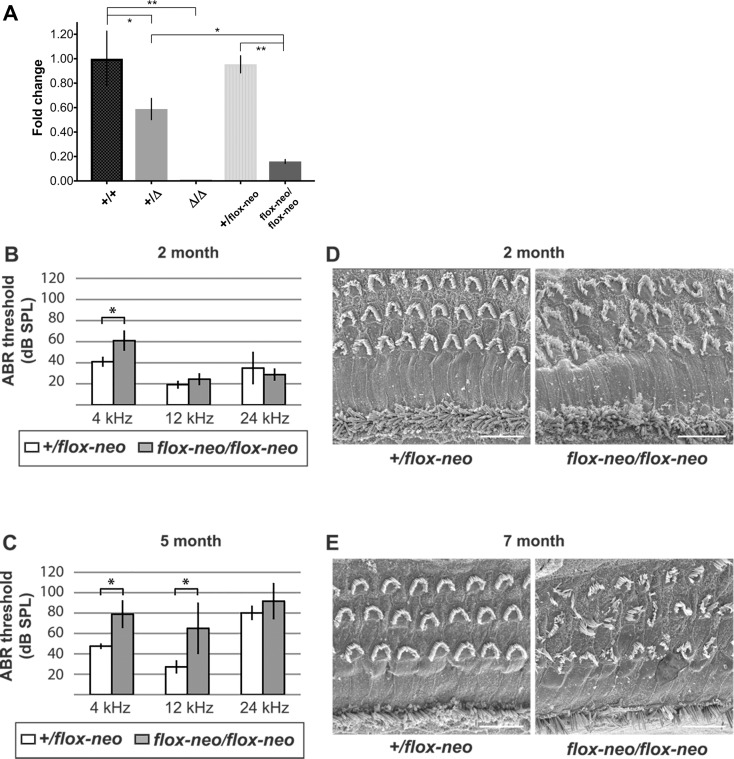
Hypomorphic expression of *Grxcr2* is associated with progressive hearing loss and stereocilia bundle defects. **(**A) *Grxcr2* transcript levels in the cochleae of +/+ mice at 3 months of age were compared by qRT-PCR to those in mice carrying either the deleted or the flox-neo alleles in heterozygous or homozygous forms (N = 2 for Δ/Δ mice; N = 3 for all other genotypes). Flox-neo/flox-neo mice expressed less than half of the level of *Grxcr2* transcripts observed in +/Δ mice. The mean fold change for Δ/Δ mice was 0.00075, SD = 0.0017. Vertical bars represent standard deviations. Asterisks represent probabilities of statistically significant differences in mean fold changes (*, p < 0.02; **, p < 0.0001). (B) At 2 months, ABR thresholds in response to 12 and 24 kHz stimuli were similar in the two genotypes whereas at 4 kHz *Grxcr2* flox-neo homozygotes had elevated hearing thresholds (p < 0.05). N = 5 mice of each genotype. (C) At 5 months, flox-neo homozygotes exhibited significant ABR threshold shifts in response to both 4 and 12 kHz stimuli (p < 0.05), indicating progression of hearing loss relative to heterozygote controls. N = 6 (+/flox) and 9 (flox-neo/flox-neo) mice. (D) Scanning electron microscopy of cochlear sensory epithelia demonstrated slight abnormalities in outer hair cell bundle orientation and organization of the flox-neo homozygotes at 2 months. (E) By 7 months, stereocilia bundles of flox-neo homozygotes exhibited extensive defects in orientation and organization. All images indicate cochlear epithelia approximately one-half turn from the apex. Similar bundle defects were observed in more basal regions of the mutant cochleae. All scale bars indicate 10 μm.

Neomycin selection cassettes in targeted alleles have been associated with decreased transcript levels of the targeted gene due to aberrant splicing from an endogenous exon into cryptic splice acceptor sites present in the neomycin cassette [60]. To test for this effect in targeted *Grxcr2*, we used qRT-PCR to compare *Grxcr2* transcript levels in the cochleae of control +/+ mice with those in mice carrying either the deleted or the flox-neo alleles in heterozygous or homozygous forms. Flox-neo/flox-neo mice expressed less than half of the level of *Grxcr2* transcripts observed in +/Δ mice, which exhibit normal hearing thresholds, consistent with a negative effect of the neo cassette and indicating the hypomorphic nature of the flox-neo allele (Fig 9A). The decreased *Grxcr2* transcript levels may be due to alternative mechanisms of interference of the neo cassette on *Grxcr2* transcription.

To identify potential effects of hypomorphic *Grxcr2* expression on auditory function, we measured ABR to pure tones in mice carrying flox-neo alleles. At two months of age, flox-neo/flox-neo mice exhibited thresholds equivalent to those of +/flox-neo mice in response to 12 and 24 kHz tones but had elevated thresholds to 4 kHz tones (Fig 9B). By five months of age, flox-neo/flox-neo mice exhibited progression of hearing loss, with increases in thresholds relative to flox-neo heterozygotes at both 4 and 12 kHz (Fig 9C). Evaluation of the apical surfaces of the organ of Corti of these mice by SEM indicated altered stereocilia bundles in a small number of outer hair cells from the *Grxcr2* flox-neo homozygotes at two months of age (Fig 9D). Consistent with the increased thresholds at later ages, nearly all outer hair cells in seven-month-old *Grxcr2* flox-neo homozygotes exhibited severe bundle defects (Fig 9E). These defects included flattened, split and misoriented bundles reminiscent of those observed in early postnatal mice homozygous for the *Grxcr2* Δ allele. These results indicate that the hypomorphic level of *Grxcr2* expression in the flox-neo homozygote mice is sufficient for relatively normal development of stereocilia bundles and hearing function at least in the more basal regions of the cochlea. This level appears to be, however, unable to sustain normal cochlear function at later ages and is associated with severe bundle defects.

### Human *GRXCR2* variant screening

Human *GRXCR2* is located on the long arm of chromosome 5 in band q32 within the candidate regions defined for two dominant, nonsyndromic hearing loss loci, *DFNA42* [61] and *DFNA54* [62]. No *GRXCR2* coding region mutations, however, have been identified in affected members of the pedigrees segregating either dominant locus (N. Gürtler and K. Xia, personal communications). We screened a large panel of probands diagnosed with congenital, severe-to-profound, nonsyndromic deafness for evidence of *GRXCR2* variants. The probands were isolated cases or from multiplex pedigrees that exhibited transmission consistent with autosomal recessive inheritance and were derived from several populations (400 Iranian cases, 139 Pakistani cases, and 87 cases from the Netherlands and mainly of Dutch origin). In the majority of probands, mutations in *GJB2*, a frequent genetic cause of hearing loss [63], had been previously excluded. In addition, given the similarity of stereocilia bundle defects in *Grxcr2* mutants with those described in mice carrying mutations in Usher genes [64], we also screened 120 index cases diagnosed with Usher syndrome, a recessive disorder that combines hearing loss with visual defects caused by retinitis pigmentosa [65]: 21 cases with type I, 47 cases with type II, 5 cases with type III, 4 cases with atypical and 43 cases with uncharacterized Usher syndrome.

We identified four *GRXCR2* nucleotide variants that result in nonsynonymous substitutions (S1 Table), all of which have been previously noted in public SNP databases. One of these (rs2569006, p.Leu181Phe) is predicted to be a neutral change based on *in silico* analyses and is also found at relatively high frequencies in many populations (≥0.1). The other three variants (rs34892428, p.Ser52Asn; rs447402, p.Glu69Lys; and rs71594518, p.Gly98Asp) occur at evolutionarily conserved positions in GRXCR2 and are predicted to be potentially deleterious. The variants rs34892428 and rs71594518, however, are also present in most populations at high frequencies (0.03 to 0.11) and so are unlikely to be pathogenic. While no frequency data was available for rs447402, it was present in probands in the heterozygous state with no other variants detected on the second allele. Alterations in *GRXCR2* are therefore unlikely to underlie hearing loss in these cohorts.

## Discussion

The phenotype of a targeted deletion mutant of *Grxcr2* described in the current study, together with localization of GRXCR2 to stereocilia, suggests GRXCR2 plays a local, intrinsic role in bundle development that influences normal orientation, organization and cohesion of the bundles. Phenotypic studies of pirouette mice that carry null mutations in *Grxcr1* [7, 66–68], demonstrated that this paralogous gene is also required for normal development of stereocilia. While GRXCR1 and GRXCR2 are similarly localized to stereocilia and loss of function mutations in each gene result in significant hearing loss at early ages, notable differences in inner ear phenotypes are associated with each mutation and suggest that GRXCR1 and GRXCR2 have distinct biological functions. Hearing loss in *Grxcr1* mutants is associated with failure of stereocilia in the cochlea to increase in diameter [67]. These early postnatal effects on stereocilia of both inner and outer hair cells are consistent with the generally more severe ABR threshold shifts observed in pirouette mice [66–68] relative to the shifts in *Grxcr2* mutants, in which early defects in bundle orientation and organization are more prominent on outer hair cells. Bundles in *Grxcr1* mutants exhibit relatively normal polarity and organization at birth, although bundles become progressively disorganized later in the first postnatal week [67]. Notably, the disorganization in pirouette is quite different in appearance from that observed in *Grxcr2* mutants (see Fig 6 in [68]) Likewise, stereocilia dimensions in *Grxcr2* mutants do not appear qualitatively different than those in control littermates, suggesting that GRXCR2 is principally involved in the determination of proper orientation and organization of the bundle.

Like many mouse deafness mutants, *Grxcr1* mutants exhibit behavioral defects such as circling and head shaking that are typical of vestibular dysfunction. This dysfunction is also associated with thin stereocilia in vestibular organs of the pirouette mutants [67]. In contrast, although GRXCR2 is normally expressed in sensory cells of vestibular organs and localized to stereocilia, *Grxcr2* Δ/Δ mutants have morphologically normal stereocilia bundles in vestibular organs, develop normal transduction currents, and do not exhibit overt behavioral defects. Adult Δ/Δ mutants do, however, exhibit higher VsEP thresholds and longer peak latencies than control mice in response to linear accelerations, indicating mild peripheral dysfunction in the utricle and/or saccule. Prolonged VsEP latencies and elevated thresholds have been described for other stereocilia mutants such as *Ptprq* [69], although dysfunction is often more severe.

The microtubule-based kinocilium plays a key role in the normal development of stereocilia bundles as it moves from its initial central location on the apical surface of the hair cell to its final, more lateral position at the vertex of the developing bundle, adjacent to the longest rank of stereocilia [70]. Several ciliary mutants that alter kinocilia formation also exhibit aberrant bundle orientation, often together with flattened bundles, indicating the importance of the kinocilium in the polarity process and in formation of the V-shaped bundle [71–74]. The kinocilia in the cochleae of early postnatal *Grxcr2* mutants were located at or very near bundle vertices, indicating that loss of *Grxcr2* function does not appear to affect production of kinocilia or this early step in polarization of the bundle.

The normal development of stereocilia bundles in the cochlea depends upon the integrated function of a diverse set of proteins, some localized within bundles and others that are located at other subcellular positions [4, 75]. Components of planar cell polarity (PCP) pathways regulate the orientation of cells and structures in a two-dimensional plane in many tissues, including the cochlea [76]. Defects in a number of PCP genes result in misorientation of bundles in the cochlea, although the organization of the bundles are otherwise normal [77–82]. The precise organization of stereocilia bundles is also dependent upon an intricate set of interstereocilia links that are first observed during early bundle development [83, 84]. The components of many of these links, as well as their associated proteins, have been identified as products of genes mutated in Usher syndrome, a sensory disorder that combines hearing loss with progressive retinal degeneration [85]. Mice that carry mutations in the genes associated with Usher syndrome type I (USH1) exhibit a combination of phenotypes, including disorganization and fragmentation of bundles as well as bundle orientation defects that are associated with mislocalized kinocilia [64]. These phenotypes indicate that interstereocilia links are required for cohesion of the developing bundles and also suggest that connecting links between the kinocilia and the tallest stereocilia are important for proper orientation of the bundle. Although not implicated in Usher syndrome, *Rac1*, which encodes a small GTPase, also appears to similarly affect bundle maturation [54].

The bundle polarity and organization defects in *Grxcr2* mutants resemble but are not identical to the phenotype of mouse USH1 mutants. Cochlear bundles in USH1 mutants exhibit severe disorganization by late embryogenesis, including fragmented and flattened bundle morphologies [64]. At early postnatal ages, bundles in *Grxcr2* mutants, although often misoriented, were relatively intact and associated with kinocilia and then underwent a rapidly progressive disorganization. The presence of intact bundles in early postnatal *Grxcr2* mutants is consistent with the presence of developmental linkages required for normal cohesion. In addition, the ability of outer hair cells in *Grxcr2* mutants to generate transduction currents suggests relatively normal assembly of the apparatus necessary for mechanotransduction, including tip links. Despite similar bundle defects, stereocilia in *Rac1* mutants also appear to acquire transduction channels normally [54], suggesting that the initial aspects of this process are independent of relative bundle orientation. The larger operating range exhibited by bundles in *Grxcr2* mutants may reflect the effects of the disorganized bundle on optimal positioning of the transduction apparatus during deflections.

The mechanism underlying the role of GRXCR2 in bundle development is unclear at present. While unlikely to possess the putative redox activity suggested for GRXCR1 [7], the central domain of GRXCR2 may retain some secondary structural characteristics of a thioredoxin fold. Thioredoxin fold domains have been implicated in roles that are independent of redox activity, including serving as interaction sites with other cellular proteins as predicted for the thioredoxin domain in the PICOT/GRX3 protein [86] and as either intrinsic [87, 88] or accessory chaperones [89]. The similarity of the C-terminal domain of GRXCR2 with Cys-rich domains present in dnaJ-related co-chaperone proteins is also consistent with this possibility. Interactions of GRXCR2 with other stereocilia components such as the Usher protein network may be required for appropriate integration of PCP and bundle cohesion/organization pathways during the course of bundle development.

The progressive hearing loss observed in mice carrying the hypomorphic flox-neo allele of *Grxcr2* suggests a potential role of the gene in maintenance of sensory function. At two months of age, homozygous flox-neo mice exhibited ABR thresholds similar to those of wild type and flox-neo heterozygote mice, suggesting that reduced levels of *Grxcr2* transcripts are sufficient for development of relatively normal function of the cochlea. Despite normal ABR thresholds, flox-neo homozygotes at two months of age exhibited a mild bundle disorganization phenotype, indicating that function of outer hair cells may be only minimally affected by such bundle defects. This situation is similar to the normal ABR thresholds and mild bundle polarization defects observed in young mice carrying mutations in the *Alms1* gene, which affect basal body/kinocilia function in cochlear hair cells [90]. Although we cannot rule out later secondary effects due to developmental abnormalities, the progressive hearing loss associated with more severe bundle defects in the older hypomorphic mutants suggests that higher levels of *Grxcr2* are required for continued cohesion and normal polarity of the bundle in the mature cochlea. The molecular control of bundle maintenance is not well understood. The association of progressive hearing loss in humans with mutations in either *MYO7A* [91] or *ACTG1* [92, 93] suggests that other genes required for stereocilia development [94, 95] may be similarly required to maintain cochlear function in the adult. Evaluation of hearing in conditional mutant mice that undergo deletion of the floxed exon 1 after auditory development and maturation should more directly clarify the role of *Grxcr2* in the mature inner ear.

Recently, a homozygous frameshift mutation in human *GRXCR2* was found to co-segregate with early onset hearing loss in a single consanguineous Pakistani pedigree, indicating the importance of the gene in human hearing and defining the *DFNB101* deafness locus [96]. The frameshift mutation is expected to result in removal of conserved residues from the C-terminus of GRXCR2 and the addition of 63 abnormal residues. Expression studies of the mutant protein in cultured cells indicated decreased stability and altered localization relative to wild type GRXCR2, consistent with a loss of function, recessive lesion. We did not detect evidence of *GRXCR2* pathogenic variants in a large screen of over 600 individuals with nonsyndromic hearing loss or in 120 individuals with Usher syndrome, suggesting that such variants are not a frequent cause of human deafness in these populations. Our demonstration of progressive hearing loss in mice carrying a hypomorphic *Grxcr2* mutation suggests that similar mutations in human could also underlie later onset deafness. Further studies of mouse *Grxcr2* mutants should provide insight into the cellular and molecular pathways that are impacted by a decrease in function of this gene during development and in the mature inner ear.

## Supporting information

S1 Fig Most non-vertebrate species contain single GRXCR homologs. **(A)** The phylogenetic tree was generated from alignment of GRXCR-related protein sequences derived from non-vertebrate lineages with mouse GRXCR1 and GRXCR2 sequences. The majority of non-vertebrate species exhibited only a single GRXCR-related protein. The tree supports a closer relationship of these proteins to mouse GRXCR1 and is consistent with the derivation of GRXCR2 homologs following duplication of an ancestral GRXCR1-like gene during early vertebrate evolution. Two species (marked with *) exhibited multiple homologs, each of which is also more closely related to mouse GRXCR1, and likely arose from lineage-specific gene duplications. Scale bar represents relative distance based upon the inferred number of substitutions per site. **(B)** The COBALT-generated sequence alignments of the C-terminal regions of GRXCR1 and GRXCR2 with those from non-vertebrate species representative of each of the eight phyla shown in (A) highlight the higher level of similarity of the non-vertebrate homologs with GRXCR1 relative to GRXCR2. Black bar, thioredoxin-like domain; bracket, position of putative GRXCR1 active site. Asterisks, conserved bipartite arrangement of cysteine residues. Gray shading indicates positions at which residues from a majority of species are identical in sequence.
(TIF)

S2 Fig Independently targeted *Grxcr2* allele exhibits hearing loss. At 5 weeks of age, *Grxcr2* Δ/Δ mutants derived from an independently targeted ES cell line (n = 3) exhibited increased ABR thresholds in response to pure tones at 4, 12, 24 and 48 kHz relative to heterozygous +/Δ (n = 3) littermates. Vertical bars represent standard deviations. Asterisks represent probabilities of statistically significant differences in threshold means (*, p < 0.02; **, p < 0.001).
(TIF)

S3 Fig *Grxcr2* mutants also exhibit auditory defects on a C57BL/6 x FVB/NJ F2 hybrid genetic background. *Grxcr2* Δ/Δ mice derived on a C57BL6/J background, which carries the *Cdh23*^*753A*^ allele (sensitivity to age-related hearing loss), were mated to FVB/NJ strain mice, which carry the *Cdh23*^*753G*^ allele (resistance to age-related hearing loss). Heterozygotes were intercrossed and F2 progeny were evaluated for *Grxcr2* and *Cdh23* genotypes. At 12 weeks of age, *Grxcr2* Δ/Δ mutants of both *Cdh23* genotypes (*Cdh23*^*753G/A*^ (n = 4) and *Cdh23*^*753A/A*^ (n = 3)) exhibited increased ABR thresholds in response to pure tones at 4, 12, and 24 kHz relative to +/+ littermates (*Cdh23*^*753G/A*^ (n = 2) and *Cdh23*^*753A/A*^ (n = 2)). All *Cdh23*^*753A/A*^ mice exhibited large threshold shifts at 48 kHz regardless of *Grxcr2* genotype, while thresholds of *Cdh23*^*753G/A*^ mice at this high frequency were elevated in Δ/Δ mutants relative to +/+ littermates. *Cdh23*^*753*^ genotypes were determined by *MspI* digestion of PCR products amplified from genomic DNA using primers derived from sequences flanking exon 7 (5’-AAAAGCCTGCAGCATTAGGA-3’; 5’-ATATGCGTGGGTGTTCACAA-3’). Vertical bars represent standard deviations.
(TIF)

S4 Fig *Grxcr2* mutants exhibit outer hair cell dysfunction. At 4 weeks of age, *Grxcr2* homozygotes (n = 5) exhibited significantly reduced DPOAE in response to 24 kHz stimuli, relative to DPOAE of heterozygous littermates (n = 5) (*, p < 0.05).
(TIF)

S5 Fig *Grxcr2* mutants exhibit normal vestibular organ morphology. SEM imaging of sensory epithelium of vestibular organs (utricles) from mice at three months of age indicated stereocilia bundle morphology and organization were comparable in *Grxcr2* heterozygotes (+/Δ) and mutant homozygotes (Δ/Δ). Scale bars indicate 5 μm.
(TIF)

S6 Fig Mechanosensitivity is preserved in neonatal utricular hair cells of homozygous *Grxcr2* mutant mice. (A) Differential interference contrast images of a P6 utricle shows preserved hair bundles in a *Grxcr2* Δ/Δ mutant.
(B) FM1-43 uptake in the same section appears normal suggesting functional mechanotransduction channels are present.
(C) Normal transduction currents are evoked in P6 +1D utricular hair cells of homozygous mutant mice. Transduction currents were recorded under the whole-cell voltage clamp mode (holding potential of -64mV).
(TIF)

S7 Fig GRXCR2 protein exhibits peak levels in cochlea stereocilia at early postnatal time points. The sensory epithelium from the cochleae of C57BL/6J mice was immunostained using an anti-GRXCR2 antibody and phalloidin to mark the stereocilia bundles. Hair cell bundles on inner and outer hair cells of mice at P0 and P3 exhibited substantial GRXCR2 reactivity while bundles from P7 mice exhibited lower reactivity, especially in outer hair cells. Images are derived from the basal and apical regions of the cochlea. Scale bars indicate 5 μm.
(TIF)

S1 Table *GRXCR2* nonsynonymous variants. (DOCX)
