# Supplementary figures and images for: Grxcr2 is required for stereocilia morphogenesis in the cochlea
Source: PLoS One. 2018 Aug 29;13(8):e0201713. doi: 10.1371/journal.pone.0201713 (PMC6114524; doi:10.1371/journal.pone.0201713)

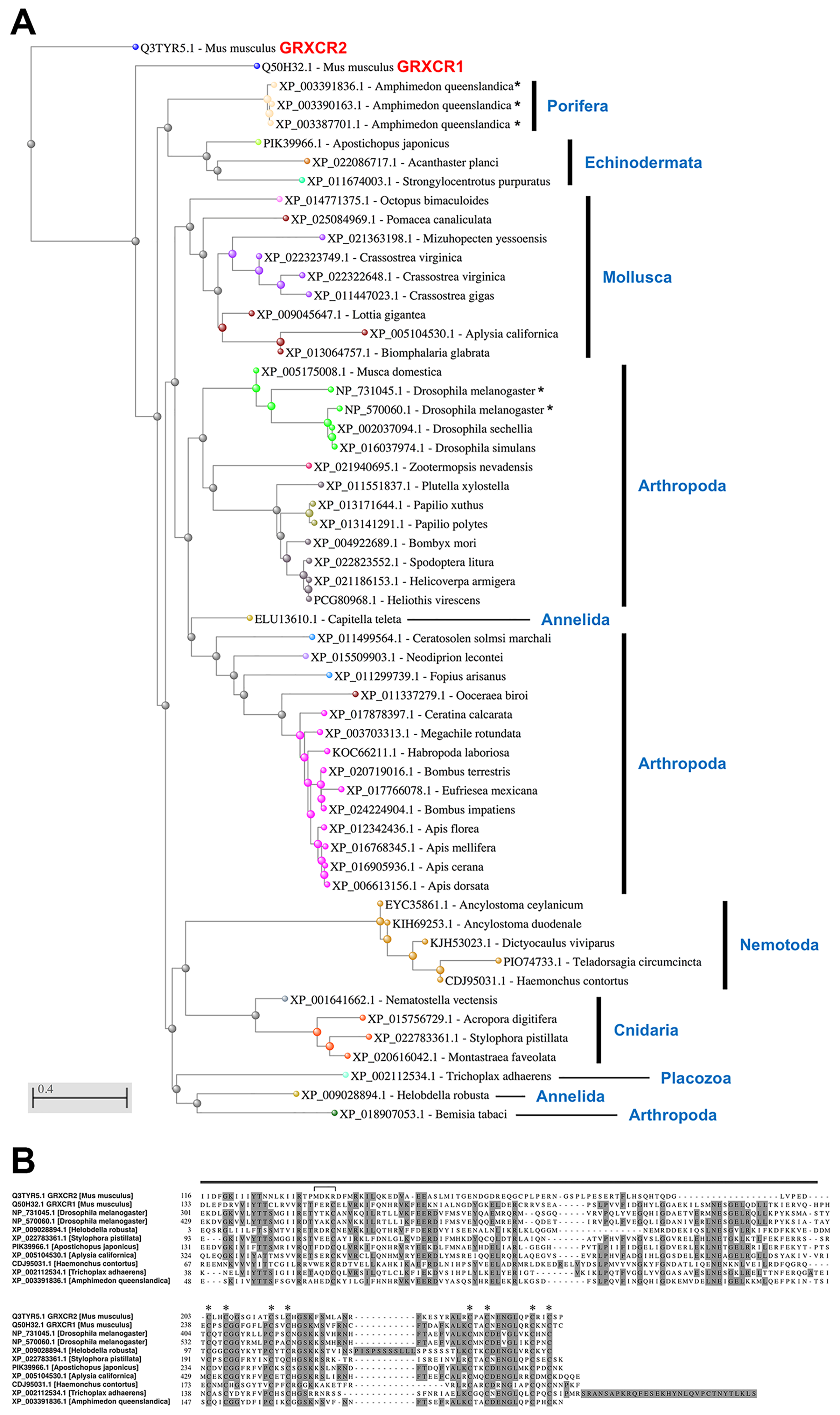

Supplement: S1 Fig — (A) The phylogenetic tree was generated from alignment of GRXCR-related protein sequences derived from non-vertebrate lineages with mouse GRXCR1 and GRXCR2 sequences. The majority of non-vertebrate species exhibited only a single GRXCR-related protein. The tree supports a closer relationship of these proteins to mouse GRXCR1 and is consistent with the derivation of GRXCR2 homologs following duplication of an ancestral GRXCR1-like gene during early vertebrate evolution. Two species (marked with *) exhibited multiple homologs, each of which is also more closely related to mouse GRXCR1, and likely arose from lineage-specific gene duplications. Scale bar represents relative distance based upon the inferred number of substitutions per site. (B) The COBALT-generated sequence alignments of the C-terminal regions of GRXCR1 and GRXCR2 with those from non-vertebrate species representative of each of the eight phyla shown in (A) highlight the higher level of similarity of the non-vertebrate homologs with GRXCR1 relative to GRXCR2. Black bar, thioredoxin-like domain; bracket, position of putative GRXCR1 active site. Asterisks, conserved bipartite arrangement of cysteine residues. Gray shading indicates positions at which residues from a majority of species are identical in sequence. (TIF) [file pone.0201713.s001.tif]

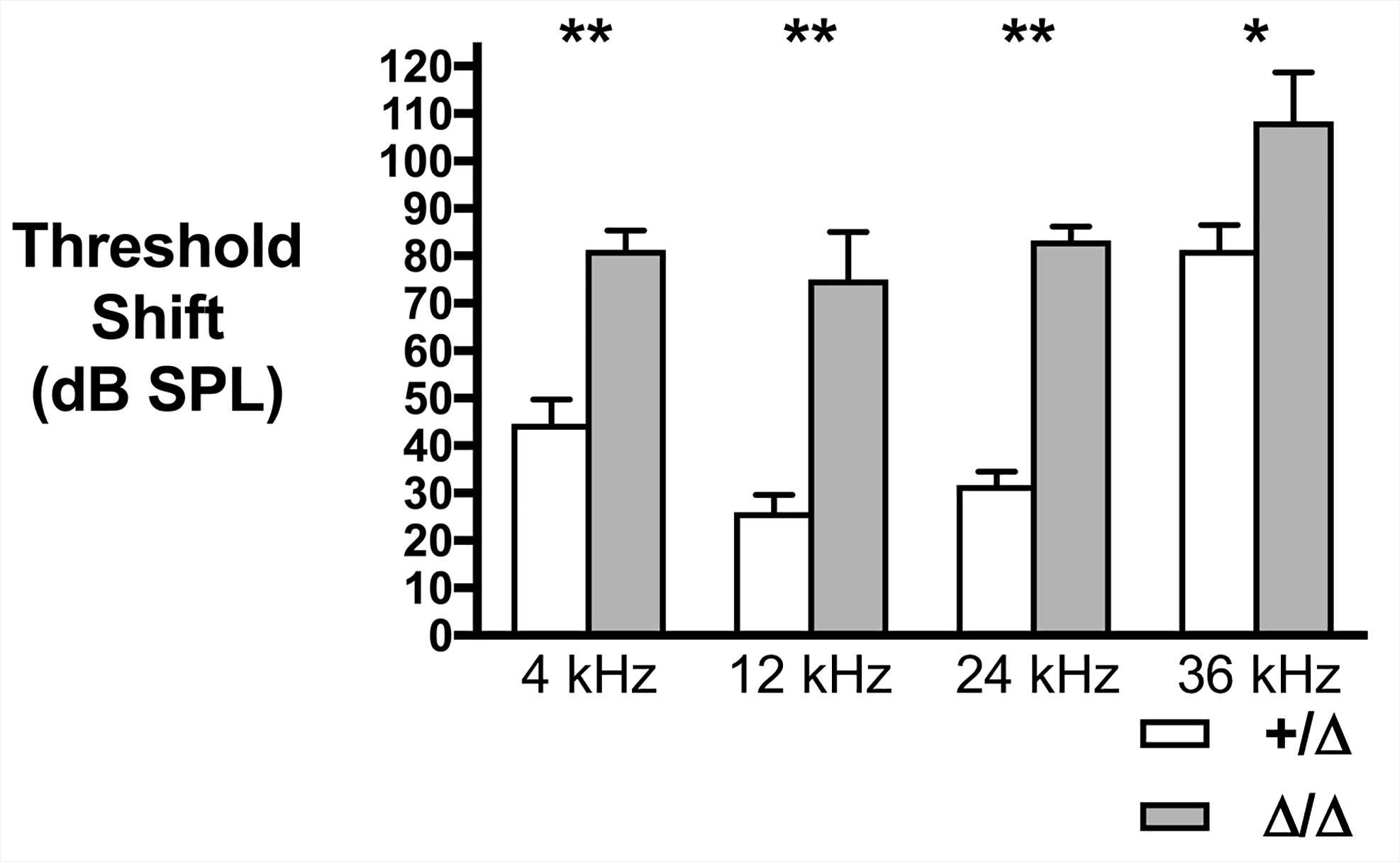

Supplement: S2 Fig — At 5 weeks of age, Grxcr2 Δ/Δ mutants derived from an independently targeted ES cell line (n = 3) exhibited increased ABR thresholds in response to pure tones at 4, 12, 24 and 48 kHz relative to heterozygous +/Δ (n = 3) littermates. Vertical bars represent standard deviations. Asterisks represent probabilities of statistically significant differences in threshold means (*, p < 0.02; **, p < 0.001). (TIF) [file pone.0201713.s002.tif]

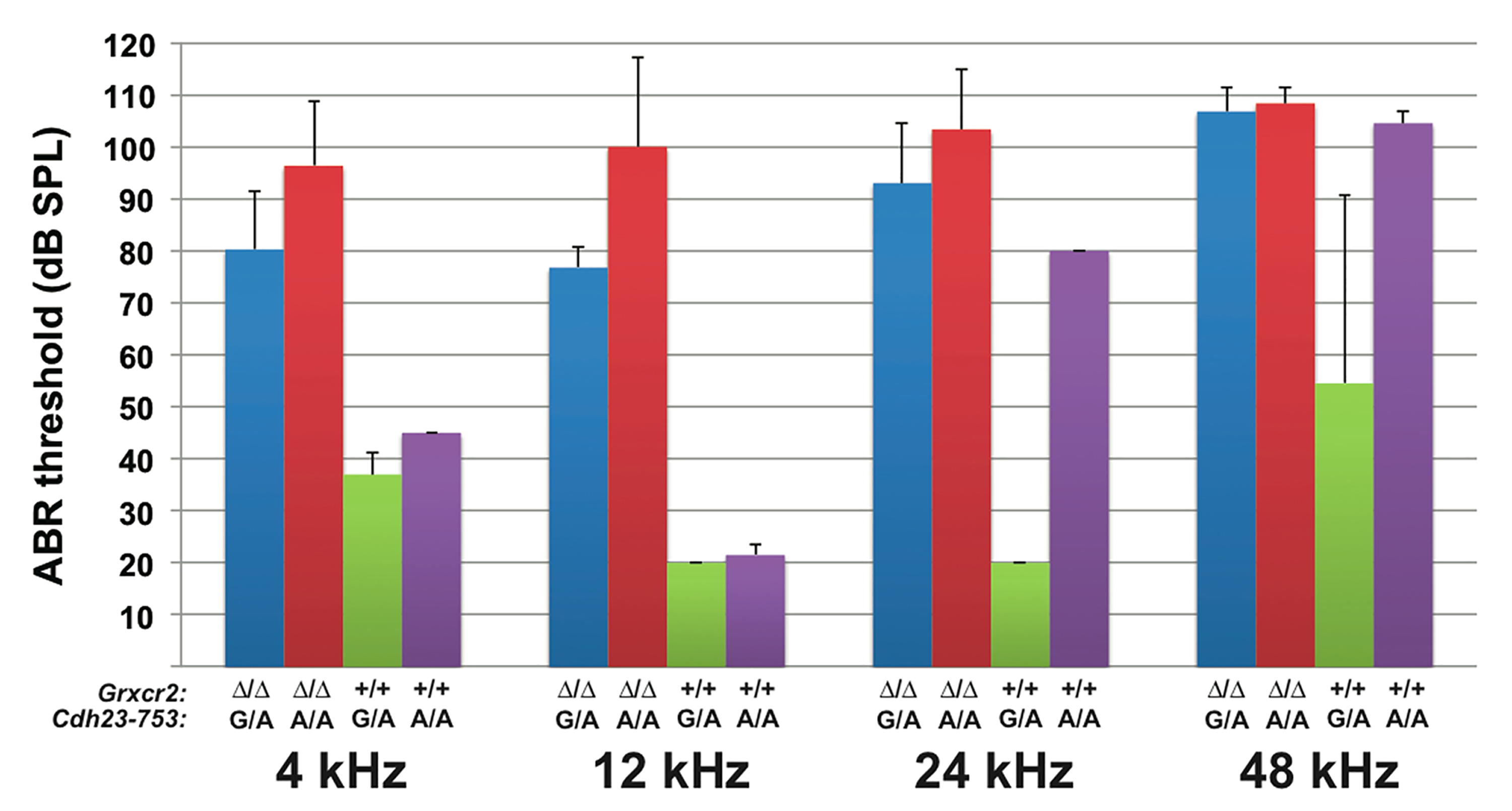

Supplement: S3 Fig — Grxcr2 Δ/Δ mice derived on a C57BL6/J background, which carries the Cdh23753A allele (sensitivity to age-related hearing loss), were mated to FVB/NJ strain mice, which carry the Cdh23753G allele (resistance to age-related hearing loss). Heterozygotes were intercrossed and F2 progeny were evaluated for Grxcr2 and Cdh23 genotypes. At 12 weeks of age, Grxcr2 Δ/Δ mutants of both Cdh23 genotypes (Cdh23753G/A (n = 4) and Cdh23753A/A (n = 3)) exhibited increased ABR thresholds in response to pure tones at 4, 12, and 24 kHz relative to +/+ littermates (Cdh23753G/A (n = 2) and Cdh23753A/A (n = 2)). All Cdh23753A/A mice exhibited large threshold shifts at 48 kHz regardless of Grxcr2 genotype, while thresholds of Cdh23753G/A mice at this high frequency were elevated in Δ/Δ mutants relative to +/+ littermates. Cdh23753 genotypes were determined by MspI digestion of PCR products amplified from genomic DNA using primers derived from sequences flanking exon 7 (5’-AAAAGCCTGCAGCATTAGGA-3’; 5’-ATATGCGTGGGTGTTCACAA-3’). Vertical bars represent standard deviations. (TIF) [file pone.0201713.s003.tif]

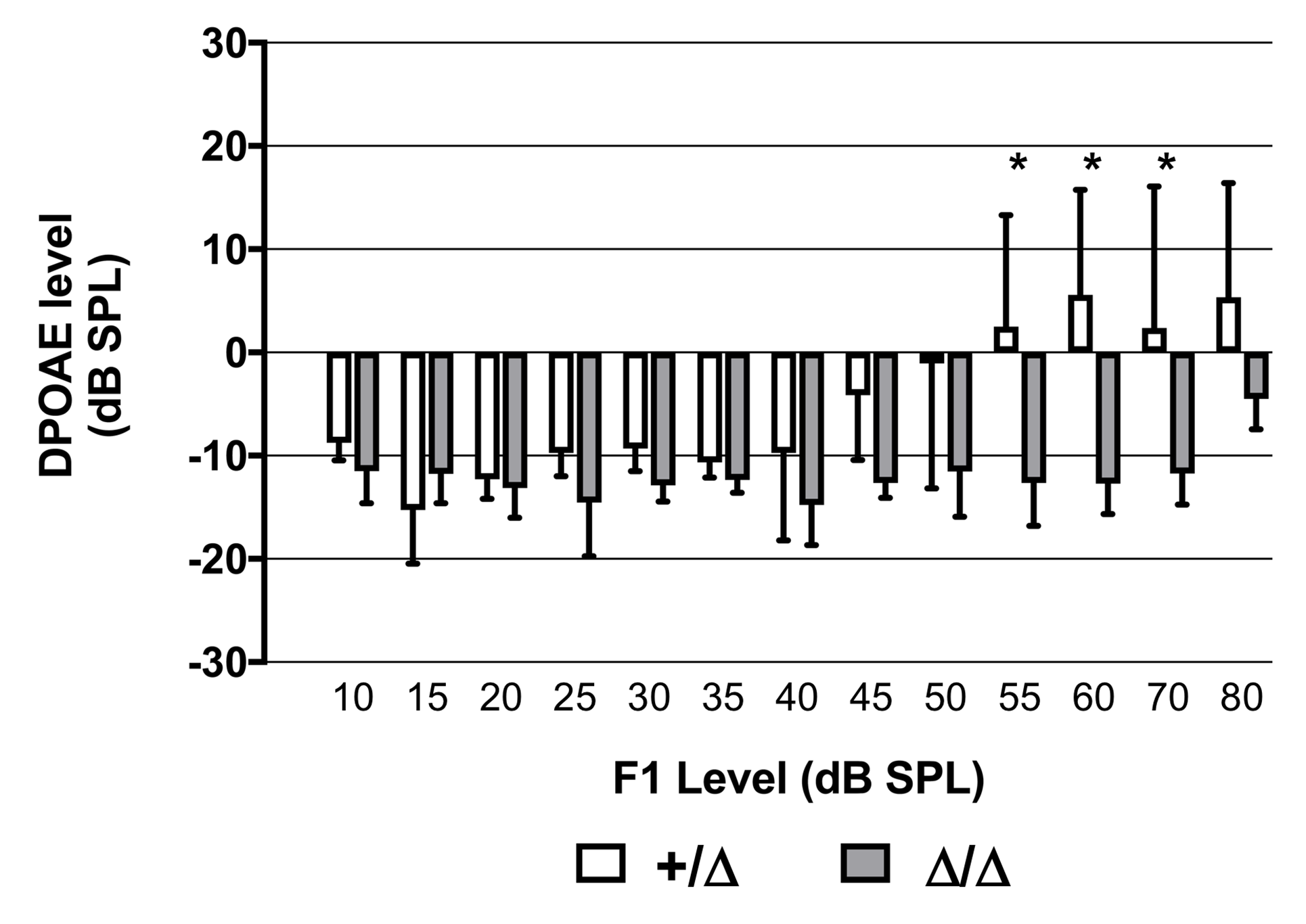

Supplement: S4 Fig — At 4 weeks of age, Grxcr2 homozygotes (n = 5) exhibited significantly reduced DPOAE in response to 24 kHz stimuli, relative to DPOAE of heterozygous littermates (n = 5) (*, p < 0.05). (TIF) [file pone.0201713.s004.tif]

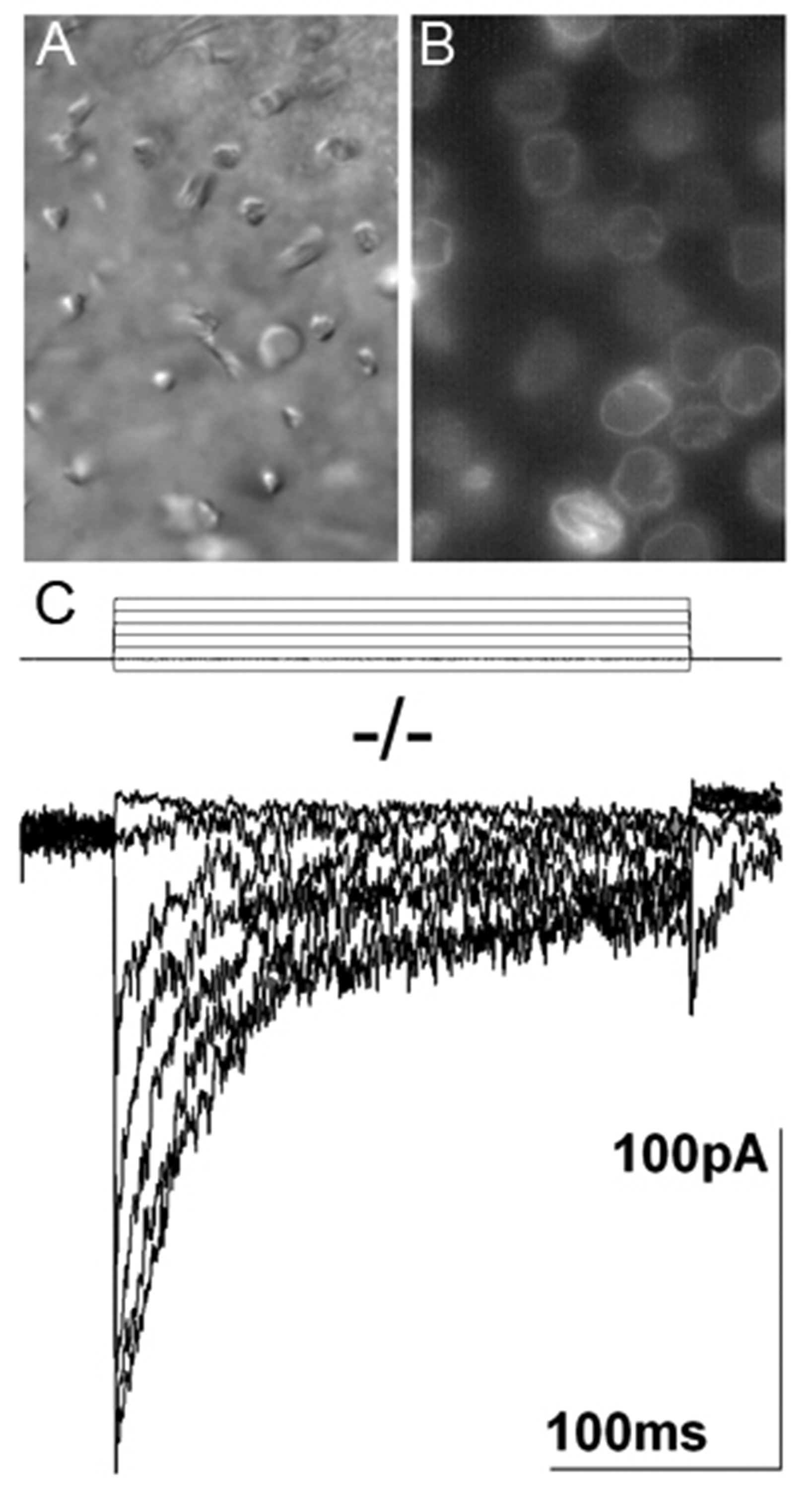

Supplement: S6 Fig — (A) Differential interference contrast images of a P6 utricle shows preserved hair bundles in a Grxcr2 Δ/Δ mutant. (B) FM1-43 uptake in the same section appears normal suggesting functional mechanotransduction channels are present. (C) Normal transduction currents are evoked in P6 +1D utricular hair cells of homozygous mutant mice. Transduction currents were recorded under the whole-cell voltage clamp mode (holding potential of -64mV). (TIF) [file pone.0201713.s006.tif]

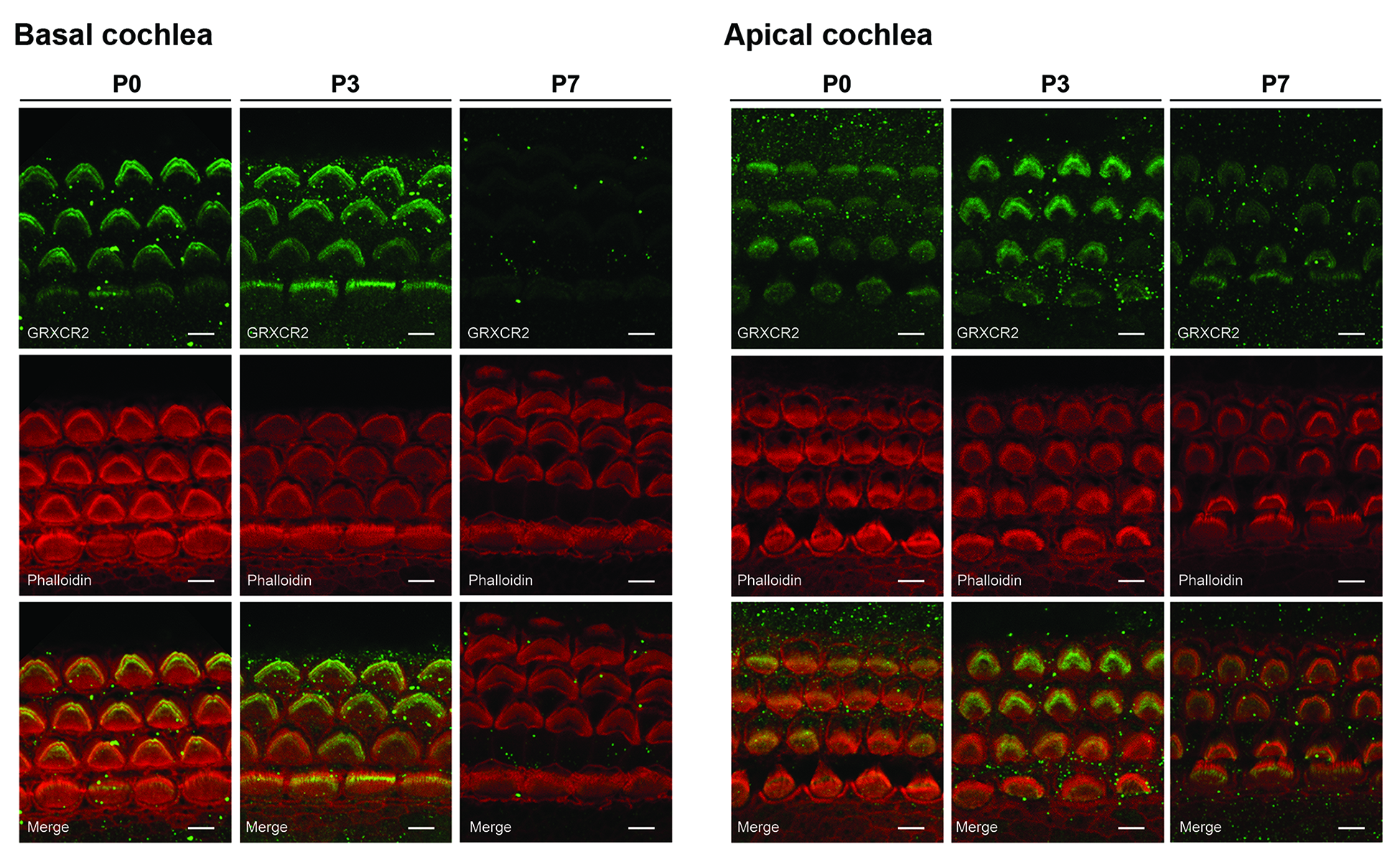

Supplement: S7 Fig — The sensory epithelium from the cochleae of C57BL/6J mice was immunostained using an anti-GRXCR2 antibody and phalloidin to mark the stereocilia bundles. Hair cell bundles on inner and outer hair cells of mice at P0 and P3 exhibited substantial GRXCR2 reactivity while bundles from P7 mice exhibited lower reactivity, especially in outer hair cells. Images are derived from the basal and apical regions of the cochlea. Scale bars indicate 5 μm. (TIF) [file pone.0201713.s007.tif]
