# Supplementary material for: Grxcr2 is required for stereocilia morphogenesis in the cochlea
Source: PLoS One. 2018 Aug 29;13(8):e0201713. doi: 10.1371/journal.pone.0201713 (PMC6114524; doi:10.1371/journal.pone.0201713)
Supplement: S1 Table — (DOCX) [file pone.0201713.s008.docx]

**Table S1. *GRXCR2* Nonsynonymous Variants**

|  | | | **Pathogenicity prediction scores** | | | | | | **Minor Allele Frequency** | | | | | | |
| --- | --- | --- | --- | --- | --- | --- | --- | --- | --- | --- | --- | --- | --- | --- | --- |
| **Variant** | **UniSNP IDs** | **aa change** | **SIFT** | **Poly phen2** | **LRT** | **Mutation Taster** | **GERP++** | **PhyloP** | **1000G overall** | **1000G AFR** | **1000G EUR** | **1000G AMR** | **1000G ASN** | **ESP6500 AA** | **ESP6500 EA** |
| chr5:145252377C>T | rs34892428 | p.S52N | T | D | D | D | C | C | 0.05 | 0.11 | 0.04 | 0.04 | 0.00 | 0.11 | 0.03 |
| chr5:145252327C>T | rs447402 | p.E69K | T | D | N | D | C | C | ND | ND | ND | ND | ND | ND | ND |
| chr5:145252239C>T | rs71594518 | p.G98D | T | D | N | N | C | C | 0.05 | 0.11 | 0.04 | 0.04 | 0.00 | 0.11 | 0.03 |
| chr5:145246085T>G | rs2569006 | p.L181F | T | B | N | N | N | N | 0.30 | 0.10 | 0.42 | 0.31 | 0.30 | 0.16 | 0.42 |

SIFT: T = tolerated; PolyPhen2: D = probably damaging, B = benign; LRT: D = Deleterious, N = Neutral; Mutation Taster: D = disease_causing, N = polymorphism; GERP++:  N = not conserved, C = conserved; PhyloP: N = not conserved, C = conserved

1000 Genomes Project data (1000G): AFR=African-American population; EUR=European-American population; ASN= Asian population

NHLBI Exome Sequencing Project data (ESP6500): AA=African-American population; EA= European-American population

ND = Not determined
